# An English-Language adaptation and validation of the Justice Sensitivity Short Scales–8 (JSS-8)

**DOI:** 10.1371/journal.pone.0293748

**Published:** 2023-11-06

**Authors:** Katharina Groskurth, Constanze Beierlein, Désirée Nießen, Anna Baumert, Beatrice Rammstedt, Clemens M. Lechner

**Affiliations:** 1 Department of Survey Design and Methodology, GESIS–Leibniz Institute for the Social Sciences, Mannheim, Germany; 2 Department Hamm 2, Hamm-Lippstadt University of Applied Sciences, Hamm, Germany; 3 Institute for Psychology, School of Human and Social Sciences, University of Wuppertal, Wuppertal, Germany; 4 Research Group on Moral Courage, Max Planck Institute for Research on Collective Goods, Bonn, Germany; University of Belgrade Faculty of Philosophy: Univerzitet u Beogradu Filozofski Fakultet, SERBIA

## Abstract

The construct of justice sensitivity has four perspectives that capture individual differences in the strength of reactions to injustice when becoming a victim of injustice (victim sensitivity), when witnessing injustice as an outsider (observer sensitivity), when passively benefitting from an injustice done to others (beneficiary sensitivity), or when committing an injustice (perpetrator sensitivity). Individual differences in these four justice sensitivity perspectives are highly relevant in moral research. With just eight items in total, the Justice Sensitivity Short Scales–8 (JSS-8) are a very efficient way to measure the four perspectives. JSS-8 was initially constructed in German (Ungerechtigkeitssensibilität-Skalen–8, USS-8) and later translated into English. In the present study, we empirically validated this English-language adaptation in a heterogeneous quota sample from the UK. The results show that the psychometric properties (i.e., reliability, validity, standardization) of JSS-8 are good, and that they are comparable with those of the German-language source version. Because of the invariance of loadings, intercepts, and residual variances, researchers can compare manifest scale statistics (i.e., means, variances) of JSS-8 across the UK and Germany. JSS-8 is thus particularly suitable for measuring justice sensitivity in various research areas with constraints on assessment time and questionnaire space.

## Introduction

Questions of fairness and justice play a central role not only in people’s private lives but also at their workplaces and in society as a whole. Numerous disciplines, such as psychology, sociology, political science, and economics, investigate justice-related issues. The significance of justice in human lives is highlighted by several influential theories in the social sciences (e.g., relative deprivation theory, equity theory, justice motive theory; for an overview, see, e.g., [1]). The desire for justice is considered a fundamental human motive [2]. Research endeavors aim to shed light on the origins, structures, and consequences of justice and injustice [3]. Since the beginning of the 21st century, justice-related research in psychology has experienced a steep increase, particularly evident in the growth in the number of publications over time [4].

To enable justice-related research under severe time constraints (e.g., in multi-thematic surveys), Baumert, Beierlein, et al. [5] and Beierlein et al. [6] developed and validated the German-language Ungerechtigkeitssensibilität-Skalen–8 (USS-8; literal translation: Injustice Sensitivity Scales–8). With eight items in total, the instrument measures four perspectives of justice sensitivity: victim, observer, beneficiary, and perpetrator sensitivity. USS-8 has proved to be a promising scale with good psychometric properties. Baumert, Beierlein, and colleagues [5] translated the German-language USS-8 into English. However, the validation of the English-language version, the Justice Sensitivity Short Scales–8 (JSS-8), remained a desideratum. The present study aimed to fill this gap by validating JSS-8 in a heterogeneous quota sample from the United Kingdom (UK), thereby enabling the instrument to be used outside the German-language context. We have entitled the English-language version “Justice Sensitivity Short Scales–8” rather than “Injustice Sensitivity Scales–8” (the literal translation of the title of the German-language scale) due to the tradition in the literature: In the English-language literature, the construct has been typically called “justice sensitivity” [7]. In the German-language literature, the construct has been commonly called “Ungerechtigkeitssensibilität” [6] or “Sensibilität für Ungerechtigkeit” [8], where the literal translation is “injustice sensitivity.” As we validated the English-language version of the scale, we decided to call the construct and label the scale according to the English-language tradition.

## Theoretical background

Justice sensitivity is an indicator of an individual’s concern for justice [9]. It is a personality trait that captures an individual’s readiness to perceive injustice and their cognitive, emotional, and behavioral reactions to it [10]. Schmitt et al. [11] initially proposed four specific components of justice sensitivity and, accordingly, four different indicators for assessing it: (a) *Frequency of experienced injustice*: Because highly justice-sensitive individuals focus more on justice-related information, they are likely to discover more situations of injustice than are justice-insensitive individuals. (b) *Intensity of emotional reactions to injustice*: Individuals with higher justice sensitivity are likely to experience more intense emotional reactions in situations they perceive as unjust. (c) *Intrusiveness of thoughts about unjust events*: Justice-sensitive individuals ruminate more about unjust treatment than do less justice-sensitive persons. (d) *Motivation to restore justice*: Highly justice-sensitive individuals have a stronger motivation to redress injustice and to support measures to restore justice (see also [7]).

According to Schmitt and colleagues [7, 8], reactions to injustice differ in part depending on an individual’s role in an episode of injustice. A person may be involved in an unjust situation as a victim, a perpetrator, an observer, or a beneficiary. Thus, justice sensitivity encompasses four theoretically related but distinguishable *perspectives*: victim sensitivity, observer sensitivity, beneficiary sensitivity, and perpetrator sensitivity. (a) *Victim sensitivity* refers to the sensitivity of becoming a victim of injustice. The dominant emotional reaction of victims of injustice is anger. Furthermore, victims tend to strive for retaliation against and punishment of the perpetrator of injustice. Accordingly, victim sensitivity describes individual differences in emotional reactions to perceived victimization and the motivation to redress such injustice. (b) *Observer sensitivity* is related to witnessing instances of injustice from an (initially) uninvolved perspective. Observers of injustice may react with indignation and outrage in an unjust situation, and observer sensitivity captures the intensity of such reactions. (c) *Beneficiary sensitivity* refers to the perspective of someone who passively benefits from injustice. (d) *Perpetrator sensitivity* refers to the perspective of someone who perceives themselves as actively committing an injustice [12]. For the latter two perspectives, feelings of guilt are a typical emotional reaction to injustice [8]. Whether and to what extent beneficiaries and perpetrators of injustice feel guilt depends on how readily they perceive injustice from the respective perspectives and how strongly they react with guilt.

All four justice sensitivity perspectives involve the motivation to redress injustice. This motivation should result in differential behaviors for the different perspectives. Observers and beneficiaries—as well as perpetrators if they feel guilty about their wrongdoing—may wish to support or compensate the victim in an unjust situation. Accordingly, observer, beneficiary, and perpetrator sensitivity should entail the motivation to compensate a victim. Moreover, victim sensitivity and observer sensitivity usually entail a punitive motivation toward a perpetrator. Conversely, perpetrator and beneficiary sensitivity should involve the inclination to self-punish or to sacrifice own advantages to restore justice.

### Empirical findings on justice sensitivity

In line with theoretical expectations, several studies have shown that the four justice sensitivity perspectives are satisfactorily distinguishable from each other and cover different aspects of justice concerns: Higher positive correlations have been reported among observer, beneficiary, and perpetrator sensitivity, whereas smaller positive correlations have been reported between victim sensitivity and the other justice sensitivity perspectives (e.g., [7]). This resonates with other findings that suggest that observer, beneficiary, and perpetrator sensitivity reflect prosocial justice concerns, whereas victim sensitivity refers to self-related justice concerns [13, 14]. Victim sensitivity has been found to correlate positively with self-related concerns such as jealousy, Machiavellianism, interpersonal distrust, the fear of being exploited, and external locus of control [6, 7, 15]. It has been reported to correlate negatively with interpersonal trust, self-efficacy, and internal locus of control [5, 6]. Life satisfaction and optimism tend to be lower at higher levels of victim sensitivity [6]. By contrast, observer, beneficiary, and perpetrator sensitivity have been shown to correlate positively with socially desirable (and other-related) personality traits such as empathy, social responsibility, and role-taking [7, 13].

The four justice sensitivity perspectives are significant predictors of several justice-related criterion variables. Across studies, victim sensitivity has been found to be positively related to antisocial and aggressive behavior (e.g., [14–16]). For example, in studies with experimental games, victim sensitivity predicted high willingness to exploit others where expedient, less cooperation in social dilemmas, and less prosocial behavior in general [17–19]. Recently, studies conducted in different cultural contexts have complemented empirical insights into antisocial consequences of victim sensitivity [12]. Moreover, this research has consistently revealed prosocial outcomes of observer, beneficiary, and perpetrator sensitivity. For example, in a cross-cultural study on the role of justice sensitivity in cooperation conducted by Baumert et al. [20] with samples from the Philippines, Germany, and Australia, individuals with a higher level of victim sensitivity were found to be more reluctant to cooperate under the threat of exploitation. By contrast, observer, beneficiary, and perpetrator sensitivity were associated with increased cooperation under temptation.

In an extensive array of life contexts, such as personal relationships [21], work [22, 23], and political contexts [24, 25], studies have demonstrated that the justice sensitivity perspectives are relevant predictors of cognition, emotion, and behavior. For example, Rothmund et al. [26] showed that victim sensitivity was associated with a preference for populist radical-right politicians and parties in the United States and Germany. By contrast, the authors found that the other three justice sensitivity dimensions were associated with a lower likelihood of having anti-immigration attitudes and a lower likelihood of preferring populist radical-right political agents.

Furthermore, justice sensitivity has been found to differ meaningfully among sociodemographic groups (e.g., [6, 7, 27]): The prosocial or other-oriented justice sensitivity dimensions are more pronounced among women than men and in groups with higher levels of education; higher personal income and higher age are related to lower victim sensitivity. However, most of the effect sizes found have been small.

Justice sensitivity not only differs notably between sociodemographic groups but also between countries: Wu and colleagues [28] looked at collectivistic (i.e., China) and individualistic societies (i.e., Germany and Russia) and found that higher collectivism was associated with higher beneficiary sensitivity but not observer sensitivity. Thus, collectivism makes people sensitive to their own advantage but not to the suffering of others.

Schmitt and colleagues [13] found all justice sensitivity perspectives to be relatively stable personality traits. Wang et al. [29] analyzed data from twins and found that all justice sensitivity perspectives were moderately heritable. In another twin study, Eftedal and colleagues [30] identified two factors, called principled and opportunistic justice sensitivity, to be essentially heritable. Whereas principled justice sensitivity represents heightened sensitivity to injustice of victims, observers, beneficiaries, and perpetrators, opportunistic justice sensitivity refers to heightened sensitivity to seeing oneself as a victim and decreased sensitivity to seeing oneself as a perpetrator.

Justice sensitivity perspectives are personality traits that are sufficiently different from other personality factors or facets (e.g., [7, 13]). Baumert and Schmitt [10] summarized the findings of several studies on the associations between justice sensitivity and other constructs and noted that justice sensitivity cannot be reduced to the broad Big Five personality factors. However, researchers have found systematic relations between justice sensitivity and both the broad Big Five personality factors and their individual facets [7, 13]: Victim sensitivity correlated negatively with Emotional Stability and Agreeableness; perpetrator sensitivity, observer sensitivity, and beneficiary sensitivity were positively associated with (some facets of) Agreeableness.

Baumert and Schmitt [10] reported preliminary evidence (*N* = 87; Baumert, unpublished data) that justice sensitivity perspectives are not correlated with working memory capacities or crystallized intelligence, but that they are correlated to a small to moderate positive extent with fluid intelligence. Furthermore, the justice sensitivity perspectives appear to be relatively independent of other justice-related dispositions, such as the belief in a just world and attitudes toward principles of distributive justice [13].

### Scale development

Since its conceptualization as a personality trait, researchers have developed different psychometric scales to measure individual differences in justice sensitivity. In an early study, Schmitt et al. [11] proposed the first scales to measure the construct that they termed “sensitivity to befallen injustice (SBI)” or “justice sensitivity.” The authors used items that tapped into four indicators for justice sensitivity: the frequency of experiences of injustice (as a measure for the readiness to perceive injustice), the intensity of anger reactions to injustice, the intrusiveness of thoughts about injustice, and punitivity (as a measure of the motivation to punish injustice).

Drawing on this work, Schmitt et al. [13] constructed an inventory with 10-item scales to measure the three justice sensitivity perspectives victim, observer, and beneficiary sensitivity (at the time, the authors called the latter scale “perpetrator sensitivity,” although it focused on passively benefitting from injustice). Thus, the inventory had 30 items in total. Schmitt et al. [7] further extended the inventory to include a scale to measure perpetrator sensitivity, resulting in 40 items in total. Notably, the four 10-item scales captured only two of the four indicators for justice sensitivity initially proposed by Schmitt et al. [11]: the intensity of emotional reactions to injustice and the intrusiveness of thoughts about injustice. This is due to the fact after investigating the factorial structure and convergent and discriminant validity of their SBI scales, Schmitt et al. [11] concluded that anger and intrusiveness of thoughts were better indicators of justice sensitivity than the reported frequency of unjust treatments and punitivity.

As the four scales proposed by Schmitt et al. [7] proved too lengthy for use in survey research with extreme time limitations, Baumert, Beierlein, et al. [5] reduced the number of items in the German-language version of the scales and developed four ultra-short two-item scales, which they entitled the Ungerechtigkeitssensibilität-Skalen–8 (USS-8; see also [6]). Reducing the number of items meant further limiting the number of indicators for justice sensitivity directly measured by the items. Thus, the two-item scales tap only into the intensity of emotional reactions to perceived injustice and a general aspect of concern about injustice (i.e., two of the four indicators for assessing justice sensitivity proposed by [11]).

Schmitt et al. [7] empirically established the factorial structure of the justice sensitivity perspectives for the long version of the justice sensitivity scales; Baumert, Beierlein, et al. [5] did so for the ultra-short version. To date, Schmitt et al. [8] have provided several translations of the long version of the four justice sensitivity scales. The ultra-short version of the German-language justice sensitivity scales, USS-8, was translated into English by Baumert, Beierlein, et al. [5] and is entitled Justice Sensitivity Short Scales–8 (JSS-8). The translation followed the International Test Commission’s two-step procedure [31], which is aligned with the TRAPD approach (Translation, Review, Adjudication, Pretesting, and Documentation; [32]). First, two professional translators independently translated the four German-language justice sensitivity scales into their respective native dialects—either American or British English. Second, a reconciliation meeting took place where the two translations were discussed and revised by a group of experts for psychological traits, the two translators, and an expert in questionnaire translation.

Baumert et al. [20] tested the measurement invariance of the 40-item Justice Sensitivity Inventory across two (English-speaking) samples from the Philippines, an Australian sample, and a German sample. The authors established weak (i.e., metric) invariance across all samples. Further, they found strong (i.e., scalar) measurement invariance of the scales between the English-speaking samples (Philippines I and II and Australia).

## Aim of the present study

No studies to date have explored the psychometric properties of the English-language ultra-short version, JSS-8. In the present study, we aimed to fill this gap by validating the JSS-8 in a newly recruited, heterogenous quota sample and directly comparing its psychometric quality with that of the German-language source version, USS-8.

We validated JSS-8 against personality and related constructs, political constructs, and sociodemographic variables such as the highest level of educational attainment and income. Based on the correlations reported in the “Theoretical background” section, we expected that higher victim sensitivity would be associated with lower Big Five Emotional Stability and Agreeableness, lower general self-efficacy, lower internal locus of control but higher external locus of control, lower interpersonal trust, lower optimism, and lower general life satisfaction. In line with other empirical findings, we further hypothesized that higher victim sensitivity would also be associated with stronger right-wing political preferences and right-wing political ideologies such as a preference for authoritarianism.

By contrast, for the other-oriented justice sensitivity perspectives—namely, observer, beneficiary, and perpetrator sensitivity—we expected positive associations with Big Five Agreeableness. Further, we predicted negative correlations with preferences for right-wing political views and ideologies.

In addition, we hypothesized that all the justice sensitivity perspectives would be positively associated with socially desirable responses. And finally, we expected that respondents’ self-reported physical health would be unrelated to the four justice sensitivity perspectives.

In the present study, we could not validate the English- and German-language JSS-8 against other existing justice sensitivity scales or other well-researched related constructs (e.g., antisocial behavior). This was because our project also served to validate other short scales, and there was no option for specific validation scales besides the core scales of the project and some other relevant variables.

## Method

### Samples

To examine the psychometric properties of JSS-8 and its comparability with the German-language source instrument, USS-8, we assessed the two versions in web-based CASI (computer-assisted self-administered interviewing) surveys conducted in parallel in January 2018 by the online access panel provider respondi AG. The survey to validate the English-language JSS-8 was conducted in the UK; the survey to assess the German-language source version was conducted in Germany (DE). Quotas from the latest German census (2011; https://ergebnisse.zensus2011.de) were the basis for selecting the two samples, representing the adult population of the respective countries in terms of age, sex, and educational attainment. That is, we kept the quotas equal across the samples from Germany and the UK, which helped to eliminate the potential effects of third variables on our results due to a different sample composition. We restricted the samples to native speakers only and financially rewarded all participants. The participants were informed about our research goal—to investigate the quality of several questionnaires. All respondents consented to participation in an online format (by clicking a button, which indicated that they had read the consent information and agreed to participate). According to the local legislation and requirements of the institution, our study on human participants did not require review and approval by an ethics committee as we collected data without any reference to the participants’ identity. The data collection was completely anonymous. We adhered to ethical standards comparable to the 1964 Declaration of Helsinki. To evaluate the stability of justice sensitivity as measured by USS-8/JSS-8, we reassessed a subsample after approximately 3 to 4 weeks (*Mdn*_UK_ = 28 days, *Mdn*_DE_ = 20 days) in both countries.

By including only respondents who did not abort the study but rather completed the entire questionnaire, we arrived at gross sample sizes of *N* = 508 for the UK and *N* = 513 for Germany. We further cleaned the data using three well-known criteria in tandem (e.g., [33]): First, we excluded respondents whose ipsatized variance (i.e., within-person variance across items; [34, 35]) was less than 5% of the distribution in the sample. Second, we excluded respondents within the upper 2.5% of the distribution of the Mahalanobis distances in the sample (i.e., we calculated the Mahalanobis distance between a respondent’s response vector and the sample means vector; [36]). Third, we excluded respondents with an average response time of less than 1 s per item [37]. We used relatively lenient cutoffs for excluding careless respondents to prevent effects on the composition of the overall quota sample (for a discussion, see [38]). In total, we excluded about 8% of the cases in the UK and German samples, resulting in net sample sizes of *N*_UK_ = 468 (retest: *N*_UK_ = 111) and *N*_DE_ = 474 (retest: *N*_DE_ = 117). We were in the lower range with 8% careless respondents compared to 5% and 15% careless respondents commonly reported in the literature (e.g., [33]). Table 1 shows the sample characteristics and their distribution. The target and real sample sizes per quota can be found in the S1 Appendix.

**Table 1:** Sample characteristics.

	United Kingdom	Germany
*N*	468	474
Mean age in years (*SD*) [Range]	45.2 (14.5) [18–69]	44.0 (14.4) [18–69]
Proportion of women (%)	52.6	50.0
Educational attainment (%)		
Low	34.8	33.5
Intermediate	32.1	33.8
High	33.1	32.7

The educational attainment levels were as follows: low = never went to school/Skills for Life/1–4 GCSEs A*–C or equivalent (Germany: *ohne Bildungsabschluss/Hauptschulabschluss* [no educational qualifications/lower secondary leaving certificate]); intermediate = 5 or more GCSEs A*–C/vocational GCSE/GNVQ intermediate or equivalent (Germany: *Mittlere Reife* [intermediate school leaving certificate]); high = 2 or more A-levels or equivalent (Germany: *(Fach-)Hochschulreife* [higher education entrance qualification]).

### Material

#### JSS-8

JSS-8 (like USS-8) consists of eight items measuring victim sensitivity, observer sensitivity, beneficiary sensitivity, and perpetrator sensitivity with two items per justice sensitivity perspective. Table 2 shows the JSS-8 items, which can also be found in a paper-and-pencil format in the S2 Appendix. The original German-language items in paper-and-pencil format can be found in the S3 Appendix as well as in Baumert, Beierlein, et al. [5] and Beierlein, Baumert, et al. [27]. JSS-8 (like USS-8) contains only positively worded items. Participants answered the items on a 6-point rating scale with labeled endpoints ranging from 1 (*does not apply at all*) to 6 (*applies completely*).

**Table 2:** Items of JSS-8.

No.	Item	Subscale
First, we will look at situations to the advantage of others and to **your own disadvantage**.		Victim sensitivity
1	It makes me angry when others are undeservingly better off than me.	
2	It worries me when I have to work hard for things that come easily to others.	
Now, we will look at situations in which you notice or learn that **someone else** is being treated unfairly, put at a disadvantage, or used.		Observer sensitivity
3	I am upset when someone is undeservingly worse off than others.	
4	It worries me when someone has to work hard for things that come easily to others.	
Now, we will look at situations that turn out **to your advantage** and to the disadvantage of others.		Beneficiary sensitivity
5	I feel guilty when I am better off than others for no reason.	
6	It bothers me when things come easily to me that others have to work hard for.	
Finally, we look at situations in which **you** treat someone else unfairly, discriminate against someone or exploit them.		Perpetrator sensitivity
7	I feel guilty when I enrich myself at the cost of others.	
8	It bothers me when I use tricks to achieve something while others have to struggle for it.	

The general instructions are as follows: “People react to unfair situations in very different ways. In the following, we would like to know how you would react in unfair situations. Please read the statements below and indicate to what extent each of these statements applies to you. If you have never experienced such a situation yourself, try to imagine how you would react if you were in such a situation.”

#### Validation measures

We validated JSS-8 against personality and related constructs, political constructs, and sociodemographic variables. We compared the findings for the English-language JSS-8 with those for the German-language USS-8. To be able to analyze the predicted associations summarized in the “Aim of the present study” section, the following scales were also part of the comprehensive online surveys:

- The extra-short form of the Big Five Inventory–2 (BFI-2-XS; English-language version: [39]; German-language version: [40]) measures the Big Five personality dimensions Extraversion, Agreeableness, Conscientiousness, Emotional Stability, and Openness with three items per dimension. The 5-point Likert scale ranges from 1 (*disagree strongly*) to 5 (*agree strongly*).
- The General Self-Efficacy Short Scale–3 (GSE-3; [41])/Allgemeine Selbstwirksamkeit Kurzskala (ASKU; [42]) comprises three items. The 5-point Likert scale ranges from 1 (*do not agree at all*) to 5 (*completely agree*).
- The Internal–External Locus of Control Short Scale–4 (IE-4; [43])/Internale–Externale-Kontrollüberzeugung–4 [44] measures internal and external locus of control with two items per factor. The 5-point Likert scale ranges from 1 (*does not apply at all*) to 5 (*applies completely*).
- The Interpersonal Trust Short Scale (KUSIV3; [45])/Kurzskala Interpersonelles Vertrauen [46] consists of three items. The 5-point Likert scale ranges from 1 (*do not agree at all*) to 5 (*completely agree*).
- The Optimism–Pessimism Short Scale–2 (SOP2; [47])/Skala Optimismus–Pessimismus–2 [48] consists of two items. The 7-point Likert scale ranges from 1 (*not at all optimistic/pessimistic*) to 7 (*very optimistic/pessimistic*).
- The General Life Satisfaction Short Scale (L-1; [49])/Kurzskala zur Erfassung der Allgemeinen Lebenszufriedenheit [50] consists of one item. The 11-point Likert scale ranges from 1 (*not at all satisfied*) to 11 (*completely satisfied*).
- The political Left–Right Self-Placement scale (English- and German-language versions: [51]) consists of one item. The 10-point Likert scale ranges from 1 (*left*) to 10 (*right*).
- The Authoritarianism Short Scale (KSA-3; [52])/Kurzskala Autoritarismus [53] measures authoritarian aggression (with four items), authoritarian submissiveness (with two items), and conventionalism (with three items). The 5-point Likert scale ranges from 1 (*do not agree at all*) to 5 (*completely agree*).
- The Social Desirability–Gamma Short Scale (KSE-G; [54])/Kurzskala Soziale Erwünschtheit–Gamma [55] measures the exaggeration of positive qualities and the minimization of negative qualities with two items each. The 5-point Likert scale ranges from 1 (*does not apply at all*) to 5 (*applies completely*).
- Self-reported physical health was measured with a single-item question used in the European Social Survey [56]. The 5-point Likert scale ranges from 1 (*very good*) to 5 (*very bad*).

Additionally, we included the following sociodemographic variables: employment (*unemployed*, 1, vs. *employed*, 2), income, educational attainment, age, and sex. We included descriptive statistics of all validation measures in the S4 Appendix.

#### Data analyses

We conducted all statistical analyses with R (version 3.6.3), using the packages lavaan [57], psych [58], and semTools [59]. We provide the analysis code in the S5 Appendix.

We estimated McDonald’s ω [60], Cronbach’s α [61], and test–retest stability over 3 to 4 weeks to investigate the reliability of the four subscales of JSS-8/USS-8 (i.e., victim, observer, beneficiary, and perpetrator sensitivity). We used Kline’s [62] heuristics to classify the reliability coefficients as adequate (.70), very good (.80), or excellent (.90).

We calculated separate confirmatory factor analysis models per country to test factorial validity. To identify the model, we fixed the first loading of each factor to 1 and the first intercept of each factor to 0. We estimated each model with robust maximum likelihood estimation (MLR). We assessed the model fit of each tested confirmatory factor analysis model via heuristics for fit indices. A model fits the data well if the confirmatory fit index (CFI) is .95 or higher, the root-mean-square error of approximation (RMSEA) is .06 or lower, and the standardized root mean residual (SRMR) is .08 or lower [63]. CFI values not lower than .90 [64], RMSEA values not higher than .10 [65], and SRMR values not higher than .10 [66] are indicative of an acceptable model fit. Furthermore, a lower Bayesian information criterion (BIC) points to a better model fit. We also report so-called robust CFI and robust RMSEA values as new robust corrections suggested by Brosseau-Liard et al. [67] and Brosseau-Liard and Savalei [68] who found that the commonly applied robust corrections of the fit indices in MLR were not theoretically justified.

We investigated the nomological network and the sociodemographic variables via correlations of manifest scale scores; thus, the correlations were lower-bound estimates of the true associations. We interpreted the correlation coefficients according to the guidelines proposed by Gignac and Szodorai [69], who interpreted correlations of .11 in individual difference research as small, correlations of .19 as medium, and correlations of .29 as large. The guidelines correspond to the 25th, 50th, and 75th percentiles of the meta-analytically derived distribution of correlations.

We used multigroup confirmatory factor analysis models [70, 71] to assess the measurement invariance of JSS-8/USS-8 across the UK and Germany. As is standard practice, we tested four successive levels of measurement invariance. First, we determined whether JSS-8 and USS-8 contained an equivalent measurement model in the UK and Germany (configural invariance). Second, we investigated whether JSS-8 and USS-8 had equivalent factor loadings (metric invariance). Third, we tested the equivalence of intercepts (scalar invariance). Fourth, we analyzed the equality of residual variances (uniqueness invariance). As outlined above for the factorial validity tests, we applied the cutoff criteria for absolute levels of fit indices proposed by Hu and Bentler [63], accompanied by the heuristics proposed by Bentler and Bonett [64], Browne and Cudeck [65], and Schermelleh-Engel et al. [66]. To evaluate the relative fit of a model, we applied Chen’s [72] cutoffs for changes in fit indices (i.e., metric invariance is rejected if ΔCFI ≤ −.010 in combination with ΔRMSEA ≥ .015 or ΔSRMR ≥ .030; scalar invariance is rejected if ΔCFI ≤ −.010 combined with ΔRMSEA ≥ .015 or ΔSRMR ≥ .010).

## Results

We analyzed descriptive statistics and psychometric properties (i.e., estimators for reliability, validity, and standardization) of the English-language JSS-8 in the UK and compared them with those of the German-language source version, USS-8, in Germany. Additionally, we conducted measurement invariance tests across both countries to evaluate test fairness.

### Descriptive statistics and reference ranges

Table 3 includes means, standard deviations, skewness, and kurtosis for JSS-8 (as well as USS-8) per subscale and item. Means of subscales and items were slightly higher in the UK than in Germany (except for Item 6). Table 3 also includes the Henze–Zirkler test to investigate multivariate normality for each subscale [73]. All values of the test were significant, indicating that multivariate normality was not given for any of the subscales. The S6 Appendix contains more detailed information on descriptive statistics and reference ranges of the subscales separately by sex and age groups.

**Table 3:** Descriptive statistics for the JSS-8 subscales and items by country.

	*M*	*SD*	Skewness	Kurtosis	HZ	*p*	MVN	ω	α	*r*_tt_
No. Item	UK	UK	UK	UK	UK	UK	UK	UK	UK	UK
	DE	DE	DE	DE	DE	DE	DE	DE	DE	DE
**Victim sensitivity**	**3.13**	**1.43**	**0.18**	**−0.84**	**8.29**	**.000**	**No**	**.80**	**.80**	**.68**
	**3.54**	**1.34**	**0.02**	**−0.63**	**4.95**	**.000**	**No**	**.78**	**.77**	**.75**
1. It makes me angry when others are undeservingly better off than me.	3.09	1.56	0.21	−1.03						
	3.46	1.53	0.03	−0.93						
2. It worries me when I have to work hard for things that come easily to others.	3.18	1.56	0.13	−1.04						
	3.62	1.44	−0.04	−0.79						
**Observer sensitivity**	**3.90**	**1.24**	**−0.26**	**−0.48**	**6.28**	**.000**	**No**	**.77**	**.76**	**.67**
	**4.01**	**1.15**	**−0.14**	**−0.32**	**8.65**	**.000**	**No**	**.74**	**.73**	**.61**
3. I am upset when someone is undeservingly worse off than others.	4.05	1.35	−0.32	−0.64						
	4.14	1.29	−0.30	−0.45						
4. It worries me when someone has to work hard for things that come easily to others.	3.75	1.40	−0.20	−0.77						
	3.88	1.30	−0.12	−0.49						
**Beneficiary sensitivity**	**2.82**	**1.34**	**0.33**	**−0.72**	**15.49**	**.000**	**No**	**.84**	**.84**	**.68**
	**2.83**	**1.35**	**0.35**	**−0.75**	**20.55**	**.000**	**No**	**.89**	**.89**	**.66**
5. I feel guilty when I am better off than others for no reason.	2.83	1.46	0.40	−0.78						
	2.89	1.47	0.32	−0.96						
6. It bothers me when things come easily to me that others have to work hard for.	2.80	1.43	0.42	−0.69						
	2.77	1.38	0.43	−0.64						
**Perpetrator sensitivity**	**3.64**	**1.63**	**−0.13**	**−1.10**	**15.80**	**.000**	**No**	**.87**	**.87**	**.46**
	**4.23**	**1.40**	**−0.49**	**−0.60**	**18.97**	**.000**	**No**	**.86**	**.86**	**.65**
7. I feel guilty when I enrich myself at the cost of others.	3.71	1.70	−0.19	−1.19						
	4.42	1.45	−0.66	−0.44						
8. It bothers me when I use tricks to achieve something while others have to struggle for it.	3.57	1.77	−0.08	−1.26						
	4.03	1.55	−0.37	−0.90						

HZ = Henze–Zirkler multivariate normality test. MVN = multivariate normality. UK = United Kingdom (*N =* 468); DE = Germany (*N =* 474). Subscale statistics are printed in bold.

### Reliability

All subscales of JSS-8 showed adequate to excellent internal consistency within the UK sample (.76 ≤ ω/α ≤ .87; see Table 3). The internal consistencies of the UK subscales were comparable with those in Germany (.73 ≤ ω/α ≤ .89). The test–retest stabilities of the JSS-8 subscales, reassessed after approximately 3 to 4 weeks, were almost adequate in the UK (.46 ≤ *r*_tt_ ≤ .68), albeit slightly lower than those in Germany (.61 ≤ *r*_tt_ ≤ .75). The test–retest stability of the perpetrator sensitivity subscale in the UK was comparatively low (*r*_tt_ = .46, all others ranged between .61 ≤ *r*_tt_ ≤ .75, see Table 3). Overall, JSS-8 (and USS-8 alike) achieved reliability estimates ranging from .61 to .89, with .46 for perpetrator sensitivity in the UK as an outlier.

### Validity

We assessed the factorial validity and nomological network of JSS-8/USS-8 to evaluate its validity.

#### Factorial validity

First, we fit a unidimensional model to test the connectedness of *all* items and to investigate whether a single underlying factor (i.e., justice sensitivity) sufficiently explains the covariance among the items. As theoretically expected, the unidimensional model did not fit well in either country, as shown in Table 4.

**Table 4:** Fit indices of different models for testing factorial validity.

Model	Fit indices									Accepted?
	χ^2^	*df*	*p*	CFI	Robust CFI	RMSEA	Robust RMSEA	SRMR	BIC	
	UK	UK	UK	UK	UK	UK	UK	UK	UK	
	DE	DE	DE	DE	DE	DE	DE	DE	DE	
1F: congeneric	605.91	20	.000	.496	.527	.250	.288	.136	13,041	No
	865.71			.248	.496	.299	.303	.136	12,695	
2F: congeneric	564.38	19	.000	.648	.672	.214	.246	.108	12,807	No
	706.79			.388	.623	.276	.269	.106	12,480	
4F^a^: congeneric	60.85	14	.000	.960	.962	.085	.098	.052	12,355	(Yes)^a^
	70.33			.950	.957	.092	.106	.040	11,936	
4F: tau-equivalent	100.06	18	.000	.929	.939	.099	.109	.055	12,370	Yes
	90.74			.935	.945	.092	.105	.043	11,936	

1F = unidimensional model, 2F = two-dimensional model, 4F = four-dimensional model.

^a^ The residual variance of Item 6 was restricted to be larger than zero in the UK.

Second, we tested a two-dimensional model, in which the items on victim sensitivity loaded on a factor separate from all other items, to investigate whether a victim sensitivity and an other-oriented factor (encompassing observer, beneficiary, and perpetrator sensitivity) sufficiently explained the covariance between items. The factors were allowed to covary. The two-dimensional model did not fit well in either country (see Table 4).

Thus, third, we tested the theoretically assumed four-dimensional confirmatory factor model. The factors were allowed to covary. However, we ran into a problem: The residual variance of Item 6 (i.e., the second beneficiary sensitivity item: “It bothers me when things come easily to me that others have to work hard for.”) was negative in the UK. When we restricted the residual variance of Item 6 in the UK to be larger than zero, we obtained an acceptable model fit in both countries (see Table 4).

The negative residual showed the instability of a model, which was just identified via covariances between latent variables. We tested an alternative model with further restrictions: Factor loadings were restricted to be the same within one latent variable (i.e., subscale). Such models are known as essentially tau-equivalent models (whereas models with freely estimated factor loadings are known as congeneric models; e.g., [74]). The essentially tau-equivalent model had an acceptable fit (see Table 4).

**Fig 1:**
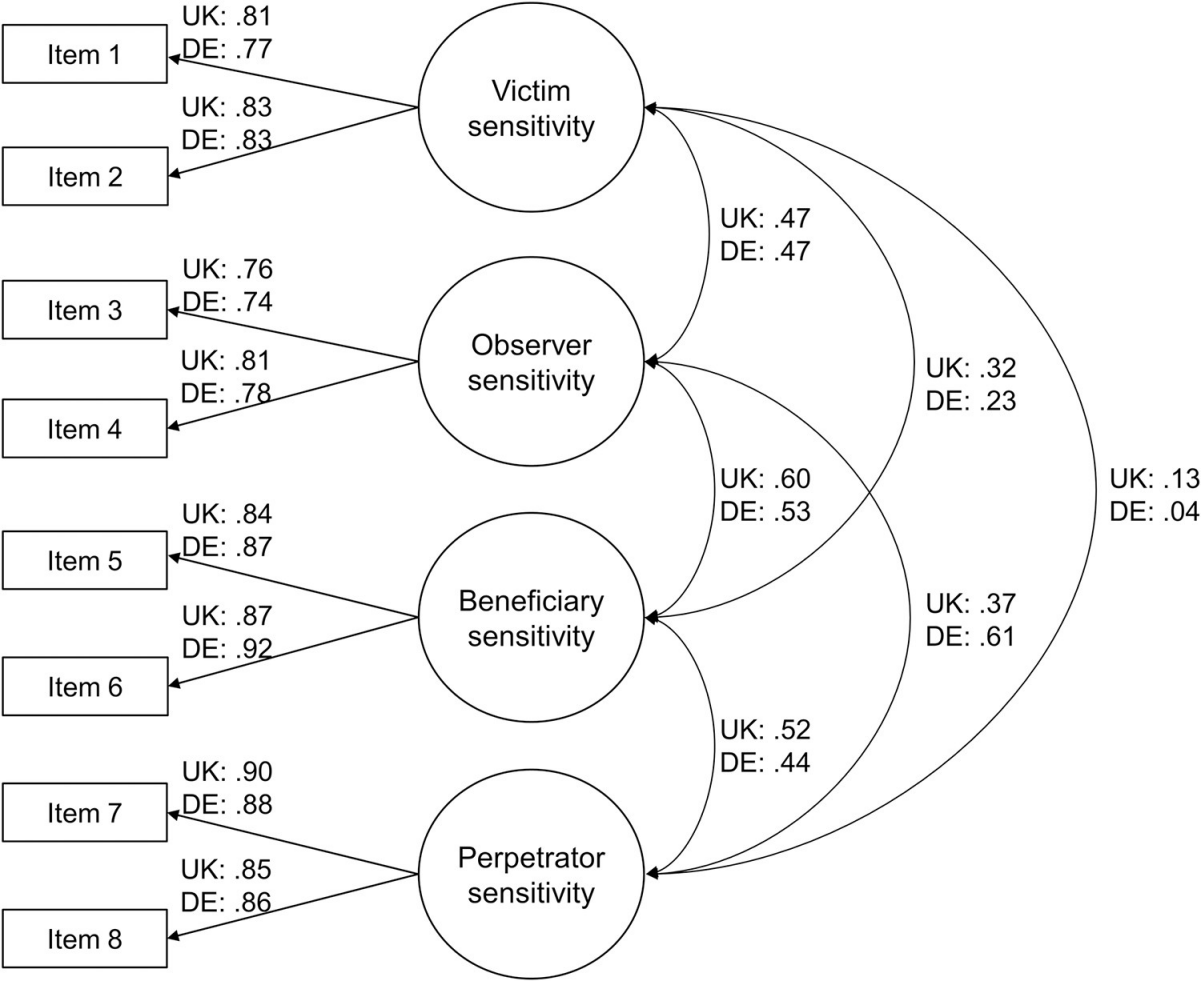
Four-factor essentially tau-equivalent measurement model of JSS-8/USS-8 with standardized coefficients. Residual terms have been omitted for clarity. *N*_UK_ = 468; *N*_DE_ = 474.

Due to the instability of the congeneric model and the still acceptable fit of the essentially tau-equivalent model, we accepted the latter one. Fig 1 shows the four-factor essentially tau-equivalent measurement model of JSS-8 (and USS-8) with standardized coefficients. We also conducted an exploratory factor analysis as a robustness check. The parallel analysis suggested four factors. Therefore, we extracted four factors by a principal axis factor analysis with oblimin rotation in both countries. All items had primary loadings as expected.

Notably, the correlations of the subscales in the essentially tau-equivalent model were consistent with theoretical expectations (see Fig 1): Observer, beneficiary, and perpetrator sensitivity (UK/DE: *r* = .37–.60/.44–.61) correlated more strongly with each other than with victim sensitivity (UK/DE: *r* = .13–.32/.04–.23). The only exception was the relatively high correlation between victim and observer sensitivity (UK and DE: *r* = .47).

#### Nomological network and sociodemographic differences

Next, we examined the nomological network and the sociodemographic correlates of JSS-8 (compared with USS-8). Table 5 shows the correlation coefficients. In the following, we highlight only correlations of at least medium size (*r* ≥ .19).

**Table 5:** Correlations of the Justice Sensitivity Scales–8 with relevant variables in the UK and German samples.

	*r* [95% CI]							
	Victim sensitivity		Observer sensitivity		Beneficiary sensitivity		Perpetrator sensitivity	
	UK	DE	UK	DE	UK	DE	UK	DE
Big Five								
Extraversion	−.01 [−.10, .08]	−.01 [−.10, .08]	−.00 [−.10, .09]	.07 [−.02, .16]	.09 [−.00, .18]	.05 [−.04, .14]	.09* [.00, .18]	.00 [−.09, .09]
Agreeableness	−.21*[−.29,−.12]	−.19*[−.28,−.10]	.12* [.03, .21]	.17* [.08, .26]	.07 [−.02, .16]	.16* [.07, .25]	.10* [.01, .19]	.25* [.16, .33]
Conscientiousness	−.09 [−.18, .00]	−.08 [−.17, .01]	−.15*[−.23,−.06]	.06 [−.03, .15]	−.27*[−.36,−.19]	−.08 [−.17, .01]	−.18*[−.26,−.09]	.06 [−.03, .15]
Emotional Stability	−.33*[−.41,−.24]	−.28*[−.36,−.19]	−.24*[−.32,−.15]	−.13*[−.22,−.04]	−.12*[−.21,−.03]	−.10*[−.19,−.01]	−.05 [−.14, .04]	−.04 [−.13, .05]
Openness	−.06 [−.15, .03]	−.04 [−.13, .05]	.14* [.05, .23]	.15* [.06, .23]	.11* [.02, .20]	.09* [.00, .18]	.15* [.06, .23]	.08 [−.01, .17]
General self-efficacy	−.06 [−.15, .03]	−.12*[−.21,−.03]	.01 [−.08, .10]	.03 [−.06, .12]	−.07 [−.16, .03]	−.10*[−.19,−.01]	.06 [−.03, .15]	.03 [−.06, .12]
Locus of control								
Internal	−.05 [−.14, .04]	−.06 [−.15, .03]	.00 [−.09, .09]	.03 [−.06, .12]	.02 [−.07, .11]	−.03 [−.12, .06]	.06 [−.03, .15]	.05 [−.04, .14]
External	.33* [.25, .41]	.25* [.17, .33]	.21* [.12, .29]	.11* [.02, .20]	.26* [.17, .34]	.11* [.02, .19]	.08 [−.02, .17]	−.05 [−.14, .04]
Interpersonal trust	−.25*[−.34,−.17]	−.30*[−.38,−.21]	.04 [−.05, .13]	.08 [−.01, .17]	.11* [.02, .20]	.04 [−.05, .13]	.15* [.06, .23]	.17* [.08, .25]
Optimism	−.26*[−.34,−.17]	−.31*[−.39,−.22]	−.05 [−.14, .04]	−.04 [−.13, .05]	−.03 [−.12, .06]	−.04 [−.13, .05]	.09 [−.00, .18]	.04 [−.05, .13]
Life satisfaction	−.26*[−.35,−.18]	−.23*[−.31,−.14]	−.13*[−.22,−.04]	−.09 [−.18, .00]	.04 [−.06, .13]	−.08 [−.17, .01]	.05 [−.04, .14]	−.02 [−.11, .07]
Left–right self-placement	.13* [.02, .24]	.10* [.00, .20]	−.14*[−.24,−.03]	−.15*[−.24,−.05]	−.05 [−.16, .06]	−.17*[−.26,−.07]	−.07 [−.18, .04]	−.15*[−.25,−.05]
Authoritarianism								
Aggression	.24* [.16, .33]	.17* [.09, .26]	−.13*[−.22,−.04]	.03 [−.06, .12]	−.08 [−.17,−.01]	−.04 [−.13, .04]	−.18*[−.27,−.09]	−.07 [−.16, .02]
Submissiveness	.19* [.11, .28]	.12* [.03, .20]	−.14*[−.23,−.05]	.02 [−.07, .11]	.11* [.02, .20]	.06 [−.03, .15]	−.08 [−.17, .01]	−.09 [−.17, .01]
Conventionalism	.12* [.03, .21]	.12* [.03, .21]	−.05 [−.14, .05]	−.00 [−.09, .09]	.02 [−.07, .11]	−.02 [−.11, .07]	−.19*[−.28,−.10]	−.13*[−.22,−.04]
Social desirability								
PQ+	−.02 [−.11, .07]	−.18*[−.26,−.09]	.20* [.11, .29]	.15* [.06, .24]	.03 [−.06, .12]	.03 [−.06, .12]	.06 [−.03, .15]	.17* [.09, .26]
NQ‒	−.29*[−.37,−.21]	−.25*[−.33,−.16]	−.00 [−.09, .09]	.14* [.06, .23]	−.27*[−.36,−.19]	−.02 [−.11, .07]	−.08 [−.17, .01]	.26* [.18, .34]
Health	−.09 [−.18, .00]	−.02 [−.11, .07]	−.12*[−.21,−.03]	−.03 [−.12, .06]	−.02 [−.11, .07]	−.01 [−.10, .08]	.09* [.00, .18]	.01 [−.08, .10]
Sociodemographic variables								
Employment	.07 [−.04, .18]	.01 [−.10, .12]	−.07 [−.17, .04]	.03 [−.08, .14]	−.03 [−.14, .07]	−.09 [−.20, .02]	.06 [−.04, .17]	.03 [−.09, .14]
Income	.03 [−.07, .12]	−.10*[−.19,−.00]	−.04 [−.14, .05]	−.08 [−.17, .02]	.06 [−.03, .15]	−.12*[−.21,−.03]	.03 [−.06, .13]	−.04 [−.14, .05]
Educational attainment	.07 [−.02, .16]	.04 [−.05, .13]	.04 [−.05, .13]	.08 [−.01, .16]	.04 [−.05, .13]	.04 [−.05, .13]	.11* [.02, .12]	.13* [.04, .22]
Age	−.32*[−.40,−.24]	−.24*[−.32,−.15]	−.11*[−.20,−.02]	−.04 [−.13, .05]	−.27*[−.36,−.19]	−.25*[−.33,−.17]	−.16*[−.24,−.07]	.02 [−.07, .11]
Sex	−.00 [−.09, .09]	.00 [−.09, .09]	.05 [−.04, .14]	.18* [.09, .27]	−.03 [−.12, .06]	.07 [−.02, .16]	−.01 [−.10, .08]	.19* [.10, .28]
Similarity of correlations across countries		.95* [.89, .98]	.74* [.47, .88]		.65* [.33, .84]		**.37** [−.05, .68]	
Similarity of correlations across subscales								
Victim sensitivity	**1.00**	**1.00**						
Observer sensitivity	**.26** [−.17, .60]	.08 [−.35, .47]	**1.00**	**1.00**				
Beneficiary sensitivity	.49* [.09, .75]	**.27** [−.16, .61]	.54* [.16, .78]	.76* [.51, .89]	**1.00**	**1.00**		
Perpetrator sensitivity	−.11 [−.50, .32]	−.53*[−.77,−.15]	.53* [.15, .77]	.72* [.44, .87]	.63* [.30, .83]	.42* [.00, .71]	**1.00**	**1.00**

UK = United Kingdom (*N =* 468; *N*_Left–right self-placement_ = 325; *N*_Employment_ = 339; *N*_Income_ = 431); DE = Germany (*N =* 474; *N*_Left–right self-placement_ = 394; *N*_Employment_ = 309; *N*_Income_ = 449); CI = confidence interval; PQ+ = exaggerating positive qualities; NQ‒ = minimizing negative qualities. Employment: 1 (*unemployed*) versus 2 (*employed*). Sex: 1 (*male*) versus 2 (*female*)

*p < .05. Medium correlations (*r* ≥ .19) are printed in bold. We recoded health and NQ‒ so that higher values correspond to higher health values and higher socially desirable responding.

In both countries, we found that higher victim sensitivity was associated with lower Agreeableness, lower Emotional Stability, lower dispositional optimism, lower interpersonal trust, lower life satisfaction, and higher external locus of control in both countries. Higher victim sensitivity was also associated with more authoritarian views in the UK. Notably, correlations of validity constructs with other-oriented justice sensitivity perspectives were often small and diverged between the two countries. We found medium to strong correlations of validity constructs with other-oriented justice sensitivity perspectives in only one of the two countries each: Lower Emotional Stability and higher external locus of control were associated with higher observer sensitivity in the UK, lower Conscientiousness and higher external locus of control was associated with higher beneficiary sensitivity in the UK, and lower conventionalism (as an authoritarian view; in the UK) and higher Agreeableness (in Germany) was associated with higher perpetrator sensitivity.

We observed that nearly all perspectives of JSS-8 (and USS-8) were affected either by exaggerating positive qualities (observer sensitivity in Germany) or by minimizing negative qualities (victim sensitivity in both countries, beneficiary sensitivity in the UK, and perpetrator sensitivity in Germany).

Correlations with sociodemographic variables and health were mainly negligible or small. We observed only medium to strong correlations of selected justice sensitivity perspectives and age: Individuals with higher values on victim sensitivity and beneficiary sensitivity were typically younger.

To quantify the cross-national similarity of correlations of the justice sensitivity perspectives with the nomological network and sociodemographic variables, we estimated Pearson correlations of all correlations across countries (see Table 5). These meta-correlations indicate that correlations across the two countries were highly similar for victim sensitivity. The correlations of observer and beneficiary sensitivity were also highly similar across countries, whereas the correlation of perpetrator sensitivity was less similar across countries.

Further, we could show that the correlation patterns with external variables are quite differentiated for all four perspectives of justice sensitivity measured by JSS-8 (and USS-8): We estimated Pearson correlations of all correlations across perspectives but within countries. Thus, within each country, we used all correlations of each justice sensitivity perspective and correlated them with those of another justice sensitivity perspective (resulting in six correlations per country, see Table 5). These meta-correlations ranged from *r* = |.11| to |.63| in the UK and from *r* = |.08| to |.76| in Germany, which shows that the perspectives have different information content and are not simply copies of each other.

### Measurement invariance across the UK and Germany

We investigated the invariance of the JSS-8/USS-8 measurement model across the UK and Germany. Measurement invariance tests were based on the essentially tau-equivalent model. Taking the essentially tau-equivalent model as a basis for measurement invariance testing, the configural and the metric model were equivalent. To be able to test a configural model against a metric model, we specified the configural model as a congeneric measurement model and the metric model as a tau-equivalent model. Fit indices are depicted in Table 6.

**Table 6:** Fit of different models testing for invariance.

	Fit indices									Accepted?
Model	χ^2^	*df*	*p*	CFI	Robust CFI	RMSEA	Robust RMSEA	SRMR	BIC	
Configural	131.15	28	.000	.955	.959	.088	.102	.046	24,332	Yes
Metric	190.47 (Δ65.30)	36 (Δ8)	.000 (Δ.000)	.932 (Δ–.023)	.942 (Δ–.017)	.095 (Δ.007)	.107 (Δ.005)	.049 (Δ.003)	24,342 (Δ10)	Yes
Scalar	206.52 (Δ14.79)	40 (Δ4)	.000 (Δ.005)	.927 (Δ–.005)	.939 (Δ–.003)	.094 (Δ–.001)	.104 (Δ–.003)	.050 (Δ.001)	24,329 (Δ–13)	Yes
Uniqueness	215.45 (Δ15.28)	48 (Δ8)	.000 (Δ.054)	.927 (Δ.000)	.936 (Δ–.003)	.086 (Δ–.008)	.098 (Δ–.006)	.050 (Δ.000)	24,299 (Δ–30)	Yes

The configural model is based on the congeneric model (with a negative residual variance of Item 6 constrained to be larger than zero in the UK); the metric, scalar, and uniqueness models are based on the essentially tau-equivalent model.

When fitting the configural model, we again encountered a negative residual of Item 6 (i.e., the second beneficiary sensitivity item) in the UK, which points to the instability of the measurement model (which is solely identified through the factor covariances). Thus, we constrained the negative residual variance to be larger than zero. The resulting configural model fit was acceptable in absolute terms (see Table 6). The fit of the metric model was substantially worse than that of the configural model according to χ^2^, CFI, and BIC. However, the metric model conflated two tests: the test of tau-equivalence, which constrains the loadings to be equal within factors, and the test of metric invariance, which constrains the loadings to be equal across factors. Therefore, we were a bit more lenient and accepted the metric model due to the negligible decrease in RMSEA and SRMR (see Table 6). The scalar model followed the metric model, and the model fit was acceptable in absolute terms. The fit of the scalar model did not deteriorate compared to the metric model (see Table 6). When the residual variances were additionally equalized across the two countries, the so-called uniqueness model fit acceptably in absolute terms. The fit was equal to the fit of the scalar model (see Table 6). Thus, the JSS-8/USS-8 measurement model and its parameters (i.e., loadings, intercepts, residual variances) were the same in the UK and Germany.

### Standardization

To facilitate objective application, JSS-8 (like USS-8) includes fixed instructions (i.e., a general instruction and specific instructions for the subscales), and the order of the items is fixed (ranging from Item 1 to 8), as is the number of labeled response options (six in total). We recommend not using a total scale score but rather building unit-weighted (i.e., unweighted) mean scores for each of the four, theoretically and empirically distinct, justice sensitivities. Thus, the instrument is accompanied by strict rules on sum score derivation to facilitate objective evaluation. Additionally, to facilitate objective interpretation, we provide reference values (i.e., descriptive statistics) in Table 3 and the S6 Appendix.

## Discussion

The ultra-short Justice Sensitivity Short Scales–8 (JSS-8) measure four aspects of justice sensitivity—victim, observer, beneficiary, and perpetrator sensitivity—with two items each. JSS-8 is the English-language adaptation of the original German-language instrument, the Ungerechtigkeitssensibilität-Skalen–8 (USS-8; [5]). The purpose of the study was to validate the English-language JSS-8 by examining its psychometric properties on a sample from the UK and comparing these with the psychometric properties of the German-language source USS-8 examined on a sample from Germany.

When looking at the descriptive statistics of JSS-8/USS-8 across countries, we found that means of victim, observer, beneficiary, and perpetrator sensitivity were only minimally higher in the UK than in Germany. It is no surprise that descriptive statistics are so similar across countries, considering the similarity of both countries based on some of Hofstede’s cultural dimensions [75]. In particular, both countries have the same index score on power distance (i.e., 35 on a scale from 1 to 100 [75]). Power distance is defined as “the extent to which the less powerful members of organizations and institutions (like the family) accept and expect that power is distributed unequally” [76]. However, the UK has a higher score (i.e., 89) than Germany (i.e., 67) on individualism. Individualism is defined as the extent to which “the ties between individuals are loose: everyone is expected to look after him/herself and his/her immediate family” [76]. Both dimensions have been shown to be related to justice perceptions in the work context (although the results of different studies were inconclusive, see [77]). Nevertheless, it can be assumed that the two dimensions also influence the general sensitivity to injustice (see also [28]). However, more research is required on the relationship between Hofstede’s cultural dimensions and justice sensitivity.

We were able to confirm the postulated four-factor structure of justice sensitivity for JSS-8 and USS-8. Correlations across subscales varied substantially; therefore, we do not recommend using a total scale score across all four justice sensitivity perspectives. Instead, unit-weighted mean scores should be computed separately for each perspective. Individual answers should be aggregated to the subscale level only if there are no missing values for any of the two items. In the case of missing data, appropriate methods for handling missing data, such as multiple imputations or full information maximum likelihood estimation, should be applied.

Moreover, cross-national measurement invariance testing revealed uniqueness invariance (i.e., same loadings, intercepts, and residual variances) for JSS-8/USS-8. Uniqueness invariance implies that researchers can compare manifest scale scores (means, variances) and correlations of JSS-8/USS-8 across Germany and the UK without introducing biases (see, e.g., [78]).

We found adequate to good reliability (i.e., internal consistency and test–retest reliability, reassessed after approximately 3 to 4 weeks) for all JSS-8/USS-8 subscales in our samples. Internal consistencies were sufficiently high for research purposes [79, 80]⸺this is especially true for short scales designed to cover the breadth of the construct [81]. Interestingly, Baumert, Beierlein, et al. [5] found similar medium to high test–retest stabilities across a similar (1-month) interval.

Whereas other researchers have found that all perspectives of justice sensitivity are relatively stable traits [13, 29, 30], the test–retest stability of the perpetrator sensitivity subscale in our study was relatively low in the UK. Further, we found that the correlations of perpetrator sensitivity with variables of the nomological network and sociodemographic variables were less similar across the UK and Germany than were the correlations of the other justice sensitivity perspectives. The instability of the perpetrator sensitivity subscale might result from the fact that respondents lacked experience of being (or perceiving themselves as) perpetrators. The instructions state that “if you have never experienced such a situation yourself, try to imagine how you would react if you were in such a situation.” It is possible that people experience victim and observer situations—and to some extent also beneficiary situations—somewhat more often than perpetrator situations. Therefore, the responses to the perpetrator items are more likely to be based on hypothetical assumptions rather than experience.

As we considered a broad range of constructs from the nomological network, several correlations between justice sensitivity perspectives (especially other-oriented ones) and variables of the nomological network were relatively low (i.e., *r* = |.00| to |.18|, similar to what has been found in other studies investigating correlations between justice sensitivity and a broad range of other constructs [27]). Still, we found several theoretically expected associations between higher victim sensitivity and lower Emotional Stability [7, 13], lower Agreeableness [7, 13], lower interpersonal trust [5, 6], lower optimism [6], lower life satisfaction [6], lower general self-efficacy [5, 6], higher external locus of control [6], and more authoritarian views [26]—all related at a medium to strong level. Unexpectedly, higher victim sensitivity was not related (or only to a small extent) to lower internal locus of control and higher right-wing political views (but the correlations tended in the expected directions [5, 6, 26]). The other-oriented justice sensitivity scales (i.e., observer, beneficiary, and perpetrator sensitivity) were associated with higher Agreeableness [7, 13]. We also expected a relation between the other-oriented justice sensitivity scales and more left-wing political preferences [26]; however, we only found negligible to small correlations for this association. Overall, these correlations suggest the distinction between the self-related, emotionally instable perspective of individuals with higher victim sensitivity and the prosocial perspective of individuals with higher other-oriented justice sensitivity. We further found that individuals with higher values on victim sensitivity and beneficiary sensitivity were typically younger [6], correlations of observer sensitivity and perpetrator sensitivity with age were negligible to small.

We observed that nearly all perspectives of JSS-8 (and USS-8) were affected by social desirability⸺a typical phenomenon with self-reporting data [82]. The use of a social desirability scale (e.g., KSE-G [48]) can control for social desirability bias in assessing response patterns.

### Limitations

In the present study, we did not validate the English- and German-language JSS-8 against other existing justice sensitivity scales or other well-researched related constructs (e.g., antisocial behavior). However, Beierlein et al. [6] provided evidence for the construct validity of the German-language source version of JSS-8 (i.e., USS-8) with the 40-item justice sensitivity scale by Schmitt and colleagues [7] from which it was derived. Moreover, the German-language source version of JSS-8 was found to be related to antisocial behavior, such as self-reported delinquency [6]. Future research could survey the English-language JSS-8 in combination with related/standard justice sensitivity scales to confirm its assumed construct validity.

It is important to note that none of the items from JSS-8/USS-8 is reverse-keyed. Thus, all items are positively pooled in relation to the underlying factor. We cannot exclude that response styles such as acquiescent responding inflate results (e.g., factor loadings, descriptive statistics, and correlations). This is not a problem specific to JSS-8/USS-8 but relevant for all scales without reverse-keyed items [83].

Further, respondents should rate the items of JSS-8/USS-8 on a 6-point Likert scale. Thus, they have no possibility to choose a neutral midpoint response. Excluding a neutral midpoint response is typically recommended when responses are likely to be influenced by social desirability [84]—such as responses to topics such as justice sensitivity, which resurfaced in the correlations examined between JSS-8/USS-8 and the social desirability scale used in the present study.

For practical reasons, we selected only the UK as the English-speaking country for our study: The online access panel provider respondi AG can recruit English-speaking participants only from the UK. However, we do not expect a different conclusion on the psychometric quality of JSS-8 in other English-speaking countries.

### Conclusion

In conclusion, the ultra-short instrument JSS-8 measured the four perspectives of justice sensitivity well across the UK and Germany. JSS-8 can now be used for research purposes in settings with extreme constraints in time or questionnaire length and in cross-national studies.

## Supporting information

S1 Appendix Quotas. (PDF)

S2 Appendix Answer sheet (English-Language version). Justice Sensitivity Short Scales–8 (JSS-8).
(PDF)

S3 Appendix Answer sheet (German-Language version). Ungerechtigkeitssensibilität-Skalen–8 (USS-8).
(PDF)

S4 Appendix Descriptive statistics for validation measures. (PDF)

S5 Appendix R analysis code. (PDF)

S6 Appendix Reference ranges. (PDF)
