# Supplementary material for: An English-Language adaptation and validation of the Justice Sensitivity Short Scales–8 (JSS-8)
Source: PLoS One. 2023 Nov 6;18(11):e0293748. doi: 10.1371/journal.pone.0293748 (PMC10627457; doi:10.1371/journal.pone.0293748)
Supplement: S1 Appendix — (PDF) [file pone.0293748.s001.pdf]

S1 Appendix: Quotas

Quotas: Target and Real Sample Sizes for the UK and Germany

Quota no.	Sex	Educational attainment	Age	Target *n* per quota	Real *n* per quota: United Kingdom	Real *n* per quota: Germany
1	male	low	18–29	22 (4.4%)	15 (3.2%)	15 (3.2%)
2			30–49	32 (6.5%)	27 (5.8%)	29 (6.1%)
3			50–69	41 (8.3%)	38 (8.1%)	40 (8.4%)
4		intermediate	18–29	21 (4.3%)	17 (3.6%)	19 (4.0%)
5			30–49	32 (6.3%)	28 (6.0%)	30 (6.3%)
6			50–69	21 (4.1%)	21 (4.5%)	24 (5.1%)
7		high	18–29	18 (3.5%)	17 (3.6%)	17 (3.6%)
8			30–49	37 (7.4%)	33 (7.1%)	37 (7.8%)
9			50–69	26 (5.1%)	26 (5.6%)	26 (5.5%)
10	female	low	18–29	16 (3.2%)	15 (3.2%)	13 (2.7%)
11			30–49	24 (4.9%)	21 (4.5%)	22 (4.6%)
12			50–69	44 (8.8%)	47 (10.0%)	40 (8.4%)
13		intermediate	18–29	22 (4.4%)	19 (4.1%)	21 (4.4%)
14			30–49	39 (7.8%)	36 (7.7%)	37 (7.8%)
15			50–69	29 (5.7%)	29 (6.2%)	29 (6.1%)
16		high	18–29	21 (4.1%)	21 (4.5%)	20 (4.2%)
17			30–49	36 (7.2%)	35 (7.5%)	36 (7.6%)
18			50–69	20 (3.9%)	23 (4.9%)	19 (4.0%)
				Σ 501 (100%)	Σ 468 (100%)	Σ 474 (100%)

Note. The educational attainment levels were as follows: low = never went to school/Skills for Life/1–4 GCSEs A*–C or equivalent (Germany: *ohne Bildungsabschluss/ Hauptschulabschluss* [no educational qualifications/lower secondary leaving certificate]); intermediate = 5 or more GCSEs A*–C/vocational GCSE/GNVQ intermediate or equivalent (Germany: *Mittlere Reife* [intermediate school leaving certificate]); high = 2 or more A-levels or equivalent (Germany: *(Fach-)Hochschulreife* [higher education entrance qualification]). The target *n* per quota was calculated based on the latest German census of 2011 (https://ergebnisse.zensus2011.de). The real *n* per quota was calculated after data cleansing.
