# Supplementary material for: An English-Language adaptation and validation of the Justice Sensitivity Short Scales–8 (JSS-8)
Source: PLoS One. 2023 Nov 6;18(11):e0293748. doi: 10.1371/journal.pone.0293748 (PMC10627457; doi:10.1371/journal.pone.0293748)
Supplement: S2 Appendix — Justice Sensitivity Short Scales–8 (JSS-8). (PDF) [file pone.0293748.s002.pdf]

S2 Appendix: Answer Sheet (English-Language Version)

Justice Sensitivity Short Scales–8 (JSS-8)

People react to unfair situations in very different ways. In the following, we would like to know how you would react in unfair situations. Please read the statements below and indicate to what extent each of these statements applies to you. If you have never experienced such a situation yourself, try to imagine how you would react if you were in such a situation.

First, we will look at situations to the advantage of others and to **your own disadvantage**.

	does not apply at all					applies completely
It makes me angry when others are undeservingly better off than me.	☐	☐	☐	☐	☐	☐
It worries me when I have to work hard for things that come easily to others.	☐	☐	☐	☐	☐	☐

Now, we will look at situations in which you notice or learn that **someone else** is being treated unfairly, put at a disadvantage, or used.

	does not apply at all					applies completely
I am upset when someone is undeservingly worse off than others.	☐	☐	☐	☐	☐	☐
It worries me when someone has to work hard for things that come easily to others.	☐	☐	☐	☐	☐	☐

Now, we will look at situations that turn out **to your advantage** and to the disadvantage of others.

	does not apply at all					applies completely
I feel guilty when I am better off than others for no reason.	☐	☐	☐	☐	☐	☐
It bothers me when things come easily to me that others have to work hard for.	☐	☐	☐	☐	☐	☐

Finally, we look at situations in which **you** treat someone else unfairly, discriminate against someone or exploit them.

	does not apply at all					applies completely
I feel guilty when I enrich myself at the cost of others.	☐	☐	☐	☐	☐	☐
It bothers me when I use tricks to achieve something while others have to struggle for it.	☐	☐	☐	☐	☐	☐
