# Supplementary material for: An English-Language adaptation and validation of the Justice Sensitivity Short Scales–8 (JSS-8)
Source: PLoS One. 2023 Nov 6;18(11):e0293748. doi: 10.1371/journal.pone.0293748 (PMC10627457; doi:10.1371/journal.pone.0293748)
Supplement: S3 Appendix — Ungerechtigkeitssensibilität-Skalen–8 (USS-8). (PDF) [file pone.0293748.s003.pdf]

Groskurth K, Beierlein C, Nießen D, Baumert A, Rammstedt B, Lechner CM. An English-language adaptation and validation of the Justice Sensitivity Short Scales–8 (JSS-8). PLOS ONE. 2023.

S3 Appendix: Answer Sheet (German-Language Version)

Ungerechtigkeitssensibilität-Skalen–8 (USS-8)

Menschen reagieren in unfairen Situationen sehr unterschiedlich. Im Folgenden möchten wir wissen, wie Sie selbst in unfairen Situationen reagieren. In den folgenden Aussagen werden verschiedene unfaire Situationen angesprochen. Bitte geben Sie an, wie sehr die jeweilige Aussage auf Sie zutrifft. Sollten Sie eine Situation noch nicht selbst erlebt haben, antworten Sie bitte so, wie Sie Ihrer Erwartung nach reagieren würden.

Zunächst geht es um Situationen, die zum Vorteil anderer und zu **Ihrem Nachteil** ausgehen.

	trifft überhaupt nicht zu					trifft voll und ganz zu
Es ärgert mich, wenn es anderen unverdient besser geht als mir.	☐	☐	☐	☐	☐	☐
Es macht mir zu schaffen, wenn ich mich für Dinge abrackern muss, die anderen in den Schoß fallen.	☐	☐	☐	☐	☐	☐

Nun geht es um Situationen, in denen Sie mitbekommen oder erfahren, dass **jemand anderes** unfair behandelt, benachteiligt oder ausgenutzt wird.

	trifft überhaupt nicht zu					trifft voll und ganz zu
Ich bin empört, wenn es jemandem unverdient schlechter geht als anderen.	☐	☐	☐	☐	☐	☐
Es macht mir zu schaffen, wenn sich jemand für Dinge abrackern muss, die anderen in den Schoß fallen.	☐	☐	☐	☐	☐	☐

Hier geht es um Situationen, die zu **Ihren Gunsten** und zum Nachteil anderer ausgehen.

	trifft überhaupt nicht zu					trifft voll und ganz zu
Ich habe Schuldgefühle, wenn es mir unverdient besser geht als anderen.	☐	☐	☐	☐	☐	☐
Es macht mir zu schaffen, wenn mir Dinge in den Schoß fallen, für die andere sich abrackern müssen.	☐	☐	☐	☐	☐	☐

Zuletzt geht es um Situationen, in denen **Sie selbst jemanden** unfair behandeln, benachteiligen oder ausnutzen.

	trifft überhaupt nicht zu					trifft voll und ganz zu
Ich habe Schuldgefühle, wenn ich mich auf Kosten anderer bereichere.	☐	☐	☐	☐	☐	☐
Es macht mir zu schaffen, wenn ich mir durch Tricks Dinge verschaffe, für die sich andere abrackern müssen.	☐	☐	☐	☐	☐	☐
