# Supplementary material for: An English-Language adaptation and validation of the Justice Sensitivity Short Scales–8 (JSS-8)
Source: PLoS One. 2023 Nov 6;18(11):e0293748. doi: 10.1371/journal.pone.0293748 (PMC10627457; doi:10.1371/journal.pone.0293748)
Supplement: S4 Appendix — (PDF) [file pone.0293748.s004.pdf]

S4 Appendix: Descriptive Statistics for Validation Measures

Descriptive Statistics for the Validation Measures by Country

	M	*SD*	Skewness	Kurtosis	HZ	*p*	MVN	Min	Max	ω	α	r_{tt}
Validation measures	UK<br>DE	UK<br>DE	UK<br>DE	UK<br>DE	UK<br>DE	UK<br>DE	UK<br>DE	UK<br>DE	UK<br>DE	UK<br>DE	UK<br>DE	UK<br>DE
**Big Five**												
Extraversion	2.80	0.87	0.06	−0.46	3.42	.000	No	1	5	.56	.57	–
	2.87	0.70	0.05	−0.01	3.26	.000	No	1	5	.36	.42	
Agreeableness	3.76	0.75	−0.43	−0.12	8.25	.000	No	1.33	5	.57	.50	–
	3.62	0.65	−0.12	−0.26	3.34	.000	No	1.67	5	.46	.37	
Conscientiousness	3.73	0.83	−0.17	−0.82	9.29	.000	No	1.33	5	.61	.57	–
	3.71	0.74	−0.09	−0.49	7.12	.000	No	1.67	5	.61	.55	
Emotional Stability	2.95	1.07	0.02	−0.78	4.71	.000	No	1	5	.79	.79	–
	3.21	0.86	−0.28	−0.30	2.78	.000	No	1	5	.71	.68	
Openness	3.39	0.77	−0.22	0.19	2.81	.000	No	1	5	.41	.44	–
	3.30	0.78	−0.06	−0.25	2.06	.000	No	1	5	.44	.46	
**General self-efficacy**	3.78	0.94	−0.71	−0.18	31.38	.000	No	1	5	.91	.91	–
	3.99	0.67	−0.50	0.56	49.16	.000	No	1.33	5	.86	.86	
**Locus of control**												
Internal	3.40	1.08	−0.29	−0.70	6.95	.000	No	1	5	.59	.59	–
	4.02	0.71	−0.84	0.97	24.49	.000	No	1	5	.68	.67	
External	2.46	0.98	0.63	−0.06	6.15	.000	No	1	5	.63	.63	–
	2.52	0.88	0.61	0.22	5.19	.000	No	1	5	.59	.59	
**Interpersonal trust**	3.20	0.81	−0.38	−0.04	8.19	.000	No	1	5	.69	.67	–
	3.07	0.79	−0.16	−0.18	7.64	.000	No	1	5	.75	.73	
**Optimism**	4.41	1.41	−0.39	−0.20	7.92	.000	No	1	7	.68	.68	–
	4.64	1.36	−0.35	−0.43	6.54	.000	No	1	7	.77	.77	
**Life satisfaction**	7.18	2.50	−0.69	−0.28	–	–	–	1	11	–	–	.82[a]
	7.40	2.44	−0.84	0.12				1	11			.71[a]
**Left–right self-placement**	5.54	2.40	−0.08	−0.69	–	–	–	1	10	–	–	–
	5.28	2.11	−0.12	−0.52				1	10			
**Authoritarianism**												
Aggression	3.24	0.95	−0.04	−0.50	3.38	.000	No	1	5	.84	.83	–
	3.29	0.89	−0.18	−0.38	2.62	.000	No	1	5	.79	.79	
Submissiveness	2.31	1.03	0.62	−0.10	16.41	.000	No	1	5	.84	.84	–
	2.53	0.99	0.24	−0.49	12.10	.000	No	1	5	.84	.83	
Conventionalism	3.02	0.93	−0.04	−0.39	5.14	.000	No	1	5	.82	.81	–
	2.98	0.93	−0.12	−0.31	3.92	.000	No	1	5	.82	.80	
**Social desirability**												
PQ+	3.55	0.73	−0.23	−0.13	5.65	.000	No	1	5	.67	.65	–
	3.67	0.68	−0.20	−0.36	8.04	.000	No	2	5	.70	.70	
NQ–	4.10	0.93	−1.29	1.07	27.13	.000	No	1	5	.79	.79	–
	3.88	0.89	−0.87	0.48	11.45	.000	No	1	5	.69	.69	
**Health**	3.54	0.92	−0.42	0.01	–	–	–	1	5	–	–	–
	3.36	0.93	−0.11	−0.52				1	5			
**Sociodemographic variables**												
Employment	1.83	0.37	−1.80	1.23	–	–	–	1	2	–	–	–
	1.93	0.26	−3.32	9.04				1	2			
Income	6.54	3.75	0.48	−0.44	–	–	–	1	17	–	–	–
	7.56	3.54	0.07	−0.76				1	17			
Educational attainment	4.74	1.88	0.00	−1.49	–	–	–	1	7	–	–	–
	4.89	2.08	0.63	−1.37				2	8			
Age	45.23	14.54	−0.03	−1.22	–	–	–	18	69	–	–	–
	43.98	14.36	−0.07	−1.10				18	69			
Sex	1.53	0.50	−0.10	−1.99	–	–	–	1	2	–	–	–
	1.50	0.50	0.00	−2.00				1	2			

Note. HZ = Henze-Zirkler multivariate normality test. MVN = multivariate normality.

UK = United Kingdom (N = 468; $N_{\text{Left–right self-placement}}$ = 325; $N_{\text{Employment}}$ = 339; N_{Income} = 431); DE = Germany (N = 474; $N_{\text{Left–right self-placement}}$ = 394; $N_{\text{Employment}}$ = 309; N_{Income} = 449).

[a] Obtained from [49].
