# Supplementary material for: An English-Language adaptation and validation of the Justice Sensitivity Short Scales–8 (JSS-8)
Source: PLoS One. 2023 Nov 6;18(11):e0293748. doi: 10.1371/journal.pone.0293748 (PMC10627457; doi:10.1371/journal.pone.0293748)
Supplement: S5 Appendix — (PDF) [file pone.0293748.s005.pdf]

S5 Appendix: R Analysis Code

```
##########
#Analysis
##########

#Clear workspace (run if desired)
rm(list = ls())

# #List of project directories (please add)
# dirs <- list(
#   data = "...",
#   analysis = "...")

#Load required package
if (!require(psych))  { install.packages("psych") } ;  library(psych)
if (!require(lavaan)) { install.packages("lavaan") } ; library(lavaan)
if (!require(semTools)) { install.packages("semTools") } ; library(semTools)
if (!require(car)) { install.packages("car") } ; library(car)
if (!require(MVN)) { install.packages("MVN") } ; library(MVN)

#Force R to not present values in exponential notion
options("scipen"= 999, "digits"=4)

#Load data
load(paste0(dirs$data, "JSS-8.rda"))

#Split data between countries
JSS8_D <- subset(JSS8, subset = (COUN == "1"))
JSS8_UK <- subset(JSS8, subset = (COUN == "2"))

########################################

#Recoding variables

#Recode the health variable so that higher values imply better health
JSS8_UK$HEAL <- 6-JSS8_UK$HEAL
JSS8_D$HEAL <- 6-JSS8_D$HEAL

#Recode the neuroticism so that higher values imply emotional stability
JSS8_UK$NEGA1 <- 6-JSS8_UK$NEGA1
JSS8_UK$NEGA2 <- 6-JSS8_UK$NEGA2
JSS8_UK$NEGA3R <- 6-JSS8_UK$NEGA3R

JSS8_D$NEGA1 <- 6-JSS8_D$NEGA1
JSS8_D$NEGA2 <- 6-JSS8_D$NEGA2
JSS8_D$NEGA3R <- 6-JSS8_D$NEGA3R

#Additionally, NQ- needs to be recoded
#so that higher values imply more socially desirable responding
JSS8_UK$SDNQ1 <- 6-JSS8_UK$SDNQ1
JSS8_UK$SDNQ2 <- 6-JSS8_UK$SDNQ2
JSS8_UK$SDNQ3 <- 6-JSS8_UK$SDNQ3
```

```
JSS8_D$SDNQ1 <- 6-JSS8_D$SDNQ1
JSS8_D$SDNQ2 <- 6-JSS8_D$SDNQ2
JSS8_D$SDNQ3 <- 6-JSS8_D$SDNQ3

################################
#Step 0: Sample Characteristics
################################

##UK

nrow(JSS8_UK)
describe(JSS8_UK$AGE)
round(describe(JSS8_UK$SEX)$mean, 3)

table(JSS8_UK$QUOT)
round((table(JSS8_UK$QUOT)/nrow(JSS8_UK))*100, 1)

edu1 <- subset(JSS8_UK, QUOT == 1 | QUOT == 2 | QUOT == 3 | QUOT == 10 | QUOT == 11 |
QUOT == 12)
edu2 <- subset(JSS8_UK, QUOT == 4 | QUOT == 5 | QUOT == 6 | QUOT == 13 | QUOT == 14 |
QUOT == 15)
edu3 <- subset(JSS8_UK, QUOT == 7 | QUOT == 8 | QUOT == 9 | QUOT == 16 | QUOT == 17 |
QUOT == 18)
round(describe(edu1$QUOT)$n/nrow(JSS8_UK)*100, 1)
round(describe(edu2$QUOT)$n/nrow(JSS8_UK)*100, 1)
round(describe(edu3$QUOT)$n/nrow(JSS8_UK)*100, 1)

##Germany

nrow(JSS8_D)
describe(JSS8_D$AGE)
round(describe(JSS8_D$SEX)$mean, 3)

table(JSS8_D$QUOT)
round((table(JSS8_D$QUOT)/nrow(JSS8_D))*100, 1)

edu1 <- subset(JSS8_D, QUOT == 1 | QUOT == 2 | QUOT == 3 | QUOT == 10 | QUOT == 11 |
QUOT == 12)
edu2 <- subset(JSS8_D, QUOT == 4 | QUOT == 5 | QUOT == 6 | QUOT == 13 | QUOT == 14 |
QUOT == 15)
edu3 <- subset(JSS8_D, QUOT == 7 | QUOT == 8 | QUOT == 9 | QUOT == 16 | QUOT == 17 |
QUOT == 18)
round(describe(edu1$QUOT)$n/nrow(JSS8_D)*100, 1)
round(describe(edu2$QUOT)$n/nrow(JSS8_D)*100, 1)
round(describe(edu3$QUOT)$n/nrow(JSS8_D)*100, 1)

################################
#Step 1: Descriptive Statistics
################################

##UK
JSS8_UK$VICT <- (JSS8_UK$VICT1+JSS8_UK$VICT2)/2
JSS8_UK$OBSE <- (JSS8_UK$OBSE1+JSS8_UK$OBSE2)/2
JSS8_UK$BENE <- (JSS8_UK$BENE1+JSS8_UK$BENE2)/2
```

```
JSS8_UK$OFFE <- (JSS8_UK$OFFE1+JSS8_UK$OFFE2)/2
describe(JSS8_UK$VICT)
describe(JSS8_UK$OBSE)
describe(JSS8_UK$BENE)
describe(JSS8_UK$OFFE)

describe(JSS8_UK$VICT1)
describe(JSS8_UK$VICT2)
describe(JSS8_UK$OBSE1)
describe(JSS8_UK$OBSE2)
describe(JSS8_UK$BENE1)
describe(JSS8_UK$BENE2)
describe(JSS8_UK$OFFE1)
describe(JSS8_UK$OFFE2)

mvn(data = JSS8_UK[, c("VICT1", "VICT2")], mvnTest = "hz")$multivariateNormality
mvn(data = JSS8_UK[, c("OBSE1", "OBSE2")], mvnTest = "hz")$multivariateNormality
mvn(data = JSS8_UK[, c("BENE1", "BENE2")], mvnTest = "hz")$multivariateNormality
mvn(data = JSS8_UK[, c("OFFE1", "OFFE2")], mvnTest = "hz")$multivariateNormality

##Germany
JSS8_D$VICT <- (JSS8_D$VICT1+JSS8_D$VICT2)/2
JSS8_D$OBSE <- (JSS8_D$OBSE1+JSS8_D$OBSE2)/2
JSS8_D$BENE <- (JSS8_D$BENE1+JSS8_D$BENE2)/2
JSS8_D$OFFE <- (JSS8_D$OFFE1+JSS8_D$OFFE2)/2
describe(JSS8_D$VICT)
describe(JSS8_D$OBSE)
describe(JSS8_D$BENE)
describe(JSS8_D$OFFE)

describe(JSS8_D$VICT1)
describe(JSS8_D$VICT2)
describe(JSS8_D$OBSE1)
describe(JSS8_D$OBSE2)
describe(JSS8_D$BENE1)
describe(JSS8_D$BENE2)
describe(JSS8_D$OFFE1)
describe(JSS8_D$OFFE2)

mvn(data = JSS8_D[, c("VICT1", "VICT2")], mvnTest = "hz")$multivariateNormality
mvn(data = JSS8_D[, c("OBSE1", "OBSE2")], mvnTest = "hz")$multivariateNormality
mvn(data = JSS8_D[, c("BENE1", "BENE2")], mvnTest = "hz")$multivariateNormality
mvn(data = JSS8_D[, c("OFFE1", "OFFE2")], mvnTest = "hz")$multivariateNormality

####################
#Step 2: Reliability
####################

##UK

#McDonald's omega & Cronbach's alpha

JSS8_MM <- 'LV_VICT =~ c(a1)*VICT1 + c(a1)*VICT2
          LV_OBSE =~ c(a2)*OBSE1 + c(a2)*OBSE2
```

```
        LV_BENE =~ c(a3)*BENE1 + c(a3)*BENE2
        LV_OFFE =~ c(a4)*OFFE1 + c(a4)*OFFE2

        VICT1+OBSE1+BENE1+OFFE1 ~ 0*1
        LV_VICT+LV_OBSE+LV_BENE+LV_OFFE ~ NA*1'

JSS8_MM.fit_UK <- sem(JSS8_MM, data = JSS8_UK, estimator = "mlr", missing = "fiml", std.lv =
FALSE)
semTools::reliability(JSS8_MM.fit_UK)

#Retest reliability

JSS8_UK$VICT <- (JSS8_UK$VICT1+JSS8_UK$VICT2)/2
JSS8_UK$OBSE <- (JSS8_UK$OBSE1+JSS8_UK$OBSE2)/2
JSS8_UK$BENE <- (JSS8_UK$BENE1+JSS8_UK$BENE2)/2
JSS8_UK$OFFE <- (JSS8_UK$OFFE1+JSS8_UK$OFFE2)/2
JSS8_UK$VICTrt <- (JSS8_UK$VICT1rt+JSS8_UK$VICT2rt)/2
JSS8_UK$OBSErt <- (JSS8_UK$OBSE1rt+JSS8_UK$OBSE2rt)/2
JSS8_UK$BENErt <- (JSS8_UK$BENE1rt+JSS8_UK$BENE2rt)/2
JSS8_UK$OFFErt <- (JSS8_UK$OFFE1rt+JSS8_UK$OFFE2rt)/2
cor.test(JSS8_UK$VICT, JSS8_UK$VICTrt, use = "pairwise.complete.obs")
cor.test(JSS8_UK$OBSE, JSS8_UK$OBSErt, use = "pairwise.complete.obs")
cor.test(JSS8_UK$BENE, JSS8_UK$BENErt, use = "pairwise.complete.obs")
cor.test(JSS8_UK$OFFE, JSS8_UK$OFFErt, use = "pairwise.complete.obs")

##########################################################################

##Germany

#McDonald's omega & Cronbach's alpha

JSS8_MM <- 'LV_VICT =~ c(a1)*VICT1 + c(a1)*VICT2
        LV_OBSE =~ c(a2)*OBSE1 + c(a2)*OBSE2
        LV_BENE =~ c(a3)*BENE1 + c(a3)*BENE2
        LV_OFFE =~ c(a4)*OFFE1 + c(a4)*OFFE2

        VICT1+OBSE1+BENE1+OFFE1 ~ 0*1
        LV_VICT+LV_OBSE+LV_BENE+LV_OFFE ~ NA*1'

JSS8_MM.fit_D <- sem(JSS8_MM, data = JSS8_D, estimator = "mlr", missing = "fiml", std.lv =
FALSE)
semTools::reliability(JSS8_MM.fit_D)

##Retest reliability

JSS8_D$VICT <- (JSS8_D$VICT1+JSS8_D$VICT2)/2
JSS8_D$OBSE <- (JSS8_D$OBSE1+JSS8_D$OBSE2)/2
JSS8_D$BENE <- (JSS8_D$BENE1+JSS8_D$BENE2)/2
JSS8_D$OFFE <- (JSS8_D$OFFE1+JSS8_D$OFFE2)/2
JSS8_D$VICTrt <- (JSS8_D$VICT1rt+JSS8_D$VICT2rt)/2
JSS8_D$OBSErt <- (JSS8_D$OBSE1rt+JSS8_D$OBSE2rt)/2
JSS8_D$BENErt <- (JSS8_D$BENE1rt+JSS8_D$BENE2rt)/2
JSS8_D$OFFErt <- (JSS8_D$OFFE1rt+JSS8_D$OFFE2rt)/2
cor.test(JSS8_D$VICT, JSS8_D$VICTrt, use = "pairwise.complete.obs")
```

```
cor.test(JSS8_D$OBSE, JSS8_D$OBSErt, use = "pairwise.complete.obs")
cor.test(JSS8_D$BENE, JSS8_D$BENErt, use = "pairwise.complete.obs")
cor.test(JSS8_D$OFFE, JSS8_D$OFFErt, use = "pairwise.complete.obs")

###########################
#Step 3: Measurement model
###########################

##UK

#EFA
psych::fa.parallel(JSS8_UK[, c("VICT1", "VICT2", "OBSE1", "OBSE2", "BENE1", "BENE2",
"OFFE1", "OFFE2")],
                   fa = "fa")$fa.values#suggests 4 factors (although the eigenvalues suggest one factor)

JSS8_UK.efa <- psych::fa(JSS8_UK[, c("VICT1", "VICT2", "OBSE1", "OBSE2", "BENE1",
"BENE2", "OFFE1", "OFFE2")],nfactors = 4,rotate = "oblimin")
print(JSS8_UK.efa, cut = .3)

#Unidimensional congeneric model
JSS8_MM1_con <- 'LV =~ VICT1 + VICT2 + OBSE1 + OBSE2 + BENE1 + BENE2 + OFFE1 +
OFFE2

                VICT1 ~ 0*1
                LV ~ NA*1'

JSS8_MM.fit <- sem(JSS8_MM1_con, data = JSS8_UK, group = "COUN", estimator = "mlr",
missing = "fiml", std.lv = FALSE)
summary(JSS8_MM.fit, standardized = TRUE, fit.measures = TRUE)

#Two-dimensional congeneric model
JSS8_MM2_con <- 'LV =~ OBSE1 + OBSE2 + BENE1 + BENE2 + OFFE1 + OFFE2
                LV_VICT =~ VICT1 + VICT2

                VICT1 + OBSE1 ~ 0*1
                LV +LV_VICT ~ NA*1'

JSS8_MM.fit <- sem(JSS8_MM2_con, data = JSS8_UK, group = "COUN", estimator = "mlr",
missing = "fiml", std.lv = FALSE)
summary(JSS8_MM.fit, standardized = TRUE, fit.measures = TRUE)

#Four-dimensional congeneric model
JSS8_MM4_con1 <- 'LV_VICT =~ VICT1 + VICT2
                 LV_OBSE =~ OBSE1 + OBSE2
                 LV_BENE =~ BENE1 + BENE2
                 LV_OFFE =~ OFFE1 + OFFE2

                 VICT1+OBSE1+BENE1+OFFE1 ~ 0*1
                 LV_VICT+LV_OBSE+LV_BENE+LV_OFFE ~ NA*1'

JSS8_MM.fit <- sem(JSS8_MM4_con1, data = JSS8_UK, group = "COUN", estimator = "mlr",
missing = "fiml", std.lv = FALSE)
summary(JSS8_MM.fit, standardized = TRUE, fit.measures = TRUE)
```

```
#...as the residual variance of OBSE2 is negative otherwise
JSS8_MM4_con2 <- 'LV_VICT =~ VICT1 + VICT2
                LV_OBSE =~ OBSE1 + OBSE2
                LV_BENE =~ BENE1 + BENE2
                LV_OFFE =~ OFFE1 + OFFE2

                VICT1+OBSE1+BENE1+OFFE1 ~ 0*1
                LV_VICT+LV_OBSE+LV_BENE+LV_OFFE ~ NA*1

                OBSE2 ~~ c(a1)*OBSE2
                a1 > 0'

JSS8_MM.fit <- sem(JSS8_MM4_con2, data = JSS8_UK, group = "COUN", estimator = "mlr",
missing = "fiml", std.lv = FALSE)
summary(JSS8_MM.fit, standardized = TRUE, fit.measures = TRUE)
# JSS8_MM.mi <- modindices(JSS8_MM.fit)
# JSS8_MM.mi[with(JSS8_MM.mi, order(-mi.scaled)), ]
#--> lots of substantial modindices

#Four-dimensional tau-equivalent model
JSS8_MM4_tau <- 'LV_VICT =~ c(a1)*VICT1 + c(a1)*VICT2
               LV_OBSE =~ c(a2)*OBSE1 + c(a2)*OBSE2
               LV_BENE =~ c(a3)*BENE1 + c(a3)*BENE2
               LV_OFFE =~ c(a4)*OFFE1 + c(a4)*OFFE2

               VICT1+OBSE1+BENE1+OFFE1 ~ 0*1
               LV_VICT+LV_OBSE+LV_BENE+LV_OFFE ~ NA*1'

JSS8_MM.fit <- sem(JSS8_MM4_tau, data = JSS8_UK, estimator = "mlr", missing = "fiml", std.lv =
FALSE)
summary(JSS8_MM.fit, standardized = TRUE, fit.measures = TRUE)
# JSS8_MM.mi <- modindices(JSS8_MM.fit)

###############################################################################

##Germany

#EFA
psych::fa.parallel(JSS8_D[, c("VICT1", "VICT2", "OBSE1", "OBSE2", "BENE1", "BENE2",
"OFFE1", "OFFE2")],
                 fa = "fa")$fa.values#suggests 4 factors (although the eigenvalues suggest one factor)

JSS8_D.efa <- psych::fa(JSS8_D[, c("VICT1", "VICT2", "OBSE1", "OBSE2", "BENE1", "BENE2",
"OFFE1", "OFFE2")],nfactors = 4,rotate = "oblimin")
print(JSS8_D.efa, cut = .3)

#Unidimensional congeneric model
JSS8_MM1_con <- 'LV =~ VICT1 + VICT2 + OBSE1 + OBSE2 + BENE1 + BENE2 + OFFE1 +
OFFE2

               VICT1 ~ 0*1
               LV ~ NA*1'
```

```
JSS8_MM.fit <- sem(JSS8_MM1_con, data = JSS8_D, group = "COUN", estimator = "mlr", missing
= "fiml", std.lv = FALSE)
summary(JSS8_MM.fit, standardized = TRUE, fit.measures = TRUE)

#Two-dimensional congeneric model
JSS8_MM2_con <- 'LV =~ OBSE1 + OBSE2 + BENE1 + BENE2 + OFFE1 + OFFE2
            LV_VICT =~ VICT1 + VICT2

            VICT1 + OBSE1 ~ 0*1
            LV +LV_VICT ~ NA*1'

JSS8_MM.fit <- sem(JSS8_MM2_con, data = JSS8_D, group = "COUN", estimator = "mlr", missing
= "fiml", std.lv = FALSE)
summary(JSS8_MM.fit, standardized = TRUE, fit.measures = TRUE)


#Four-dimensional congeneric model
JSS8_MM4_con1 <- 'LV_VICT =~ VICT1 + VICT2
             LV_OBSE =~ OBSE1 + OBSE2
             LV_BENE =~ BENE1 + BENE2
             LV_OFFE =~ OFFE1 + OFFE2

             VICT1+OBSE1+BENE1+OFFE1 ~ 0*1
             LV_VICT+LV_OBSE+LV_BENE+LV_OFFE ~ NA*1'

JSS8_MM.fit <- sem(JSS8_MM4_con1, data = JSS8_D, group = "COUN", estimator = "mlr", missing
= "fiml", std.lv = FALSE)
summary(JSS8_MM.fit, standardized = TRUE, fit.measures = TRUE)

#Four-dimensional tau-equivalent model
JSS8_MM4_tau <- 'LV_VICT =~ c(a1)*VICT1 + c(a1)*VICT2
            LV_OBSE =~ c(a2)*OBSE1 + c(a2)*OBSE2
            LV_BENE =~ c(a3)*BENE1 + c(a3)*BENE2
            LV_OFFE =~ c(a4)*OFFE1 + c(a4)*OFFE2

            VICT1+OBSE1+BENE1+OFFE1 ~ 0*1
            LV_VICT+LV_OBSE+LV_BENE+LV_OFFE ~ NA*1'

JSS8_MM.fit <- sem(JSS8_MM4_tau, data = JSS8_D, estimator = "mlr", missing = "fiml", std.lv =
FALSE)
summary(JSS8_MM.fit, standardized = TRUE, fit.measures = TRUE)
# JSS8_MM.mi <- modindices(JSS8_MM.fit)

###########################################
#Step 4: Construct and criterion validity
###########################################

##UK

#Empty vectors to save correlations
UK_VICT_cor <- c()
UK_OBSE_cor <- c()
UK_BENE_cor <- c()
UK_OFFE_cor <- c()
```

```
dimension.matrix <- list(c("EXTR", "AGRE", "CONS", "NEGA", "OPEN", "ASKU", "ILOC",
"ELOC",
                          "KUSIV", "SOP", "LISA1", "LRPL1", "AAGG", "ASUB", "CONV",
                          "SDPQ", "SDNQ", "HEAL", "EMPL.new", "INCO", "SCHO", "AGE", "SEX"),
                        c("UK", "D"))

#############################
#Justice sensitivity (JSS-8)
#############################
JSS8_UK$VICT <- (JSS8_UK$VICT1+JSS8_UK$VICT2)/2
JSS8_UK$OBSE <- (JSS8_UK$OBSE1+JSS8_UK$OBSE2)/2
JSS8_UK$BENE <- (JSS8_UK$BENE1+JSS8_UK$BENE2)/2
JSS8_UK$OFFE <- (JSS8_UK$OFFE1+JSS8_UK$OFFE2)/2

#####################
#Big Five (BFI-2-XS)
#####################
JSS8_UK$EXTR <- (JSS8_UK$EXTR1R+JSS8_UK$EXTR2+JSS8_UK$EXTR3)/3 #Extraversion
JSS8_UK$AGRE <- (JSS8_UK$AGRE1+JSS8_UK$AGRE2R+JSS8_UK$AGRE3)/3
#Agreeableness
JSS8_UK$CONS <- (JSS8_UK$CONS1R+JSS8_UK$CONS2R+JSS8_UK$CONS3)/3
#Conscientiousness
JSS8_UK$NEGA <- (JSS8_UK$NEGA1+JSS8_UK$NEGA2+JSS8_UK$NEGA3R)/3 #Neuroticism
JSS8_UK$OPEN <- (JSS8_UK$OPEN1+JSS8_UK$OPEN2R+JSS8_UK$OPEN3)/3 #Openness

describe(JSS8_UK$EXTR)
mvn(data = JSS8_UK[, c("EXTR1R", "EXTR2", "EXTR3")], mvnTest = "hz")$multivariateNormality
describe(JSS8_UK$AGRE)
mvn(data = JSS8_UK[, c("AGRE1", "AGRE2R", "AGRE3")], mvnTest =
"hz")$multivariateNormality
describe(JSS8_UK$CONS)
mvn(data = JSS8_UK[, c("CONS1R", "CONS2R", "CONS3")], mvnTest =
"hz")$multivariateNormality
describe(JSS8_UK$NEGA)
mvn(data = JSS8_UK[, c("NEGA1", "NEGA2", "NEGA3R")], mvnTest =
"hz")$multivariateNormality
describe(JSS8_UK$OPEN)
mvn(data = JSS8_UK[, c("OPEN1", "OPEN2R", "OPEN3")], mvnTest = "hz")$multivariateNormality

BFI_MM <- 'LV_EXTR =~ EXTR1R+EXTR2+EXTR3
        LV_AGRE =~ AGRE1+AGRE2R+AGRE3
        LV_CONS =~ CONS1R+CONS2R+CONS3
        LV_NEGA =~ NEGA1+NEGA2+NEGA3R
        LV_OPEN =~ OPEN1+OPEN2R+OPEN3

        EXTR1R+AGRE1+CONS1R+NEGA1+OPEN1 ~ 0*1
        LV_EXTR+LV_AGRE+LV_CONS+LV_NEGA+LV_OPEN ~ NA*1

        AGRE2R ~~ c(a1)*AGRE2R
        a1 > 0'

BFI_MM.fit_UK <- sem(BFI_MM, data = JSS8_UK, estimator = "mlr", missing = "fiml", std.lv =
FALSE)
```

```
semTools::reliability(BFI_MM.fit_UK)


cor.test(JSS8_UK$VICT, JSS8_UK$EXTR, use = "pairwise.complete.obs")
UK_VICT_cor <- c(UK_VICT_cor, cor.test(JSS8_UK$VICT, JSS8_UK$EXTR, use =
"pairwise.complete.obs")[["estimate"]][["cor"]])
cor.test(JSS8_UK$OBSE, JSS8_UK$EXTR, use = "pairwise.complete.obs")
UK_OBSE_cor <- c(UK_OBSE_cor, cor.test(JSS8_UK$OBSE, JSS8_UK$EXTR, use =
"pairwise.complete.obs")[["estimate"]][["cor"]])
cor.test(JSS8_UK$BENE, JSS8_UK$EXTR, use = "pairwise.complete.obs")
UK_BENE_cor <- c(UK_BENE_cor, cor.test(JSS8_UK$BENE, JSS8_UK$EXTR, use =
"pairwise.complete.obs")[["estimate"]][["cor"]])
cor.test(JSS8_UK$OFFE, JSS8_UK$EXTR, use = "pairwise.complete.obs")
cor.test(JSS8_UK$OFFE, JSS8_UK$EXTR, use = "pairwise.complete.obs")$p.value #p value below
.05?
UK_OFFE_cor <- c(UK_OFFE_cor, cor.test(JSS8_UK$OFFE, JSS8_UK$EXTR, use =
"pairwise.complete.obs")[["estimate"]][["cor"]])

cor.test(JSS8_UK$VICT, JSS8_UK$AGRE, use = "pairwise.complete.obs")
UK_VICT_cor <- c(UK_VICT_cor, cor.test(JSS8_UK$VICT, JSS8_UK$AGRE, use =
"pairwise.complete.obs")[["estimate"]][["cor"]])
cor.test(JSS8_UK$OBSE, JSS8_UK$AGRE, use = "pairwise.complete.obs")
UK_OBSE_cor <- c(UK_OBSE_cor, cor.test(JSS8_UK$OBSE, JSS8_UK$AGRE, use =
"pairwise.complete.obs")[["estimate"]][["cor"]])
cor.test(JSS8_UK$BENE, JSS8_UK$AGRE, use = "pairwise.complete.obs")
UK_BENE_cor <- c(UK_BENE_cor, cor.test(JSS8_UK$BENE, JSS8_UK$AGRE, use =
"pairwise.complete.obs")[["estimate"]][["cor"]])
cor.test(JSS8_UK$OFFE, JSS8_UK$AGRE, use = "pairwise.complete.obs")
UK_OFFE_cor <- c(UK_OFFE_cor, cor.test(JSS8_UK$OFFE, JSS8_UK$AGRE, use =
"pairwise.complete.obs")[["estimate"]][["cor"]])

cor.test(JSS8_UK$VICT, JSS8_UK$CONS, use = "pairwise.complete.obs")
cor.test(JSS8_UK$VICT, JSS8_UK$CONS, use = "pairwise.complete.obs")$p.value #p value below
.05?
UK_VICT_cor <- c(UK_VICT_cor, cor.test(JSS8_UK$VICT, JSS8_UK$CONS, use =
"pairwise.complete.obs")[["estimate"]][["cor"]])
cor.test(JSS8_UK$OBSE, JSS8_UK$CONS, use = "pairwise.complete.obs")
UK_OBSE_cor <- c(UK_OBSE_cor, cor.test(JSS8_UK$OBSE, JSS8_UK$CONS, use =
"pairwise.complete.obs")[["estimate"]][["cor"]])
cor.test(JSS8_UK$BENE, JSS8_UK$CONS, use = "pairwise.complete.obs")
UK_BENE_cor <- c(UK_BENE_cor, cor.test(JSS8_UK$BENE, JSS8_UK$CONS, use =
"pairwise.complete.obs")[["estimate"]][["cor"]])
cor.test(JSS8_UK$OFFE, JSS8_UK$CONS, use = "pairwise.complete.obs")
UK_OFFE_cor <- c(UK_OFFE_cor, cor.test(JSS8_UK$OFFE, JSS8_UK$CONS, use =
"pairwise.complete.obs")[["estimate"]][["cor"]])

cor.test(JSS8_UK$VICT, JSS8_UK$NEGA, use = "pairwise.complete.obs")
UK_VICT_cor <- c(UK_VICT_cor, cor.test(JSS8_UK$VICT, JSS8_UK$NEGA, use =
"pairwise.complete.obs")[["estimate"]][["cor"]])
cor.test(JSS8_UK$OBSE, JSS8_UK$NEGA, use = "pairwise.complete.obs")
UK_OBSE_cor <- c(UK_OBSE_cor, cor.test(JSS8_UK$OBSE, JSS8_UK$NEGA, use =
"pairwise.complete.obs")[["estimate"]][["cor"]])
cor.test(JSS8_UK$BENE, JSS8_UK$NEGA, use = "pairwise.complete.obs")
```

```
UK_BENE_cor <- c(UK_BENE_cor, cor.test(JSS8_UK$BENE, JSS8_UK$NEGA, use =
"pairwise.complete.obs")[["estimate"]][["cor"]])
cor.test(JSS8_UK$OFFE, JSS8_UK$NEGA, use = "pairwise.complete.obs")
UK_OFFE_cor <- c(UK_OFFE_cor, cor.test(JSS8_UK$OFFE, JSS8_UK$NEGA, use =
"pairwise.complete.obs")[["estimate"]][["cor"]])

cor.test(JSS8_UK$VICT, JSS8_UK$OPEN, use = "pairwise.complete.obs")
UK_VICT_cor <- c(UK_VICT_cor, cor.test(JSS8_UK$VICT, JSS8_UK$OPEN, use =
"pairwise.complete.obs")[["estimate"]][["cor"]])
cor.test(JSS8_UK$OBSE, JSS8_UK$OPEN, use = "pairwise.complete.obs")
UK_OBSE_cor <- c(UK_OBSE_cor, cor.test(JSS8_UK$OBSE, JSS8_UK$OPEN, use =
"pairwise.complete.obs")[["estimate"]][["cor"]])
cor.test(JSS8_UK$BENE, JSS8_UK$OPEN, use = "pairwise.complete.obs")
UK_BENE_cor <- c(UK_BENE_cor, cor.test(JSS8_UK$BENE, JSS8_UK$OPEN, use =
"pairwise.complete.obs")[["estimate"]][["cor"]])
cor.test(JSS8_UK$OFFE, JSS8_UK$OPEN, use = "pairwise.complete.obs")
UK_OFFE_cor <- c(UK_OFFE_cor, cor.test(JSS8_UK$OFFE, JSS8_UK$OPEN, use =
"pairwise.complete.obs")[["estimate"]][["cor"]])


################################
#General self-efficacy (GSE-3)
################################
JSS8_UK$ASKU <- (JSS8_UK$ASKU1+JSS8_UK$ASKU2+JSS8_UK$ASKU3)/3

describe(JSS8_UK$ASKU)
mvn(data = JSS8_UK[, c("ASKU1", "ASKU2", "ASKU3")], mvnTest = "hz")$multivariateNormality


ASKU_MM <- 'LV =~ ASKU1+ASKU2+ASKU3

        ASKU1 ~ 0*1
        LV ~ NA*1'

ASKU_MM.fit_UK <- sem(ASKU_MM, data = JSS8_UK, estimator = "mlr", missing = "fiml", std.lv
= FALSE)
semTools::reliability(ASKU_MM.fit_UK)


cor.test(JSS8_UK$VICT, JSS8_UK$ASKU, use = "pairwise.complete.obs")
UK_VICT_cor <- c(UK_VICT_cor, cor.test(JSS8_UK$VICT, JSS8_UK$ASKU, use =
"pairwise.complete.obs")[["estimate"]][["cor"]])
cor.test(JSS8_UK$OBSE, JSS8_UK$ASKU, use = "pairwise.complete.obs")
UK_OBSE_cor <- c(UK_OBSE_cor, cor.test(JSS8_UK$OBSE, JSS8_UK$ASKU, use =
"pairwise.complete.obs")[["estimate"]][["cor"]])
cor.test(JSS8_UK$BENE, JSS8_UK$ASKU, use = "pairwise.complete.obs")
UK_BENE_cor <- c(UK_BENE_cor, cor.test(JSS8_UK$BENE, JSS8_UK$ASKU, use =
"pairwise.complete.obs")[["estimate"]][["cor"]])
cor.test(JSS8_UK$OFFE, JSS8_UK$ASKU, use = "pairwise.complete.obs")
UK_OFFE_cor <- c(UK_OFFE_cor, cor.test(JSS8_UK$OFFE, JSS8_UK$ASKU, use =
"pairwise.complete.obs")[["estimate"]][["cor"]])

#############################################
#Internal-external locus of control (IE-4)
```

```
##########################################
JSS8_UK$ILOC <- (JSS8_UK$ILOC1+JSS8_UK$ILOC2)/2
JSS8_UK$ELOC <- (JSS8_UK$ELOC1+JSS8_UK$ELOC2)/2

describe(JSS8_UK$ILOC)
describe(JSS8_UK$ELOC)
mvn(data = JSS8_UK[, c("ILOC1", "ILOC2")], mvnTest = "hz")$multivariateNormality
mvn(data = JSS8_UK[, c("ELOC1", "ELOC2")], mvnTest = "hz")$multivariateNormality

LOC_MM <- 'LV_ILOC =~ c(a)*ILOC1+c(a)*ILOC2
	LV_ELOC =~ c(b)*ELOC1+c(b)*ELOC2

	ILOC1+ELOC1 ~ 0*1
	LV_ILOC+LV_ELOC ~ NA*1'

LOC_MM.fit_UK <- sem(LOC_MM, data = JSS8_UK, estimator = "mlr", missing = "fiml", std.lv =
FALSE)
semTools::reliability(LOC_MM.fit_UK)

cor.test(JSS8_UK$VICT, JSS8_UK$ILOC, use = "pairwise.complete.obs")
UK_VICT_cor <- c(UK_VICT_cor, cor.test(JSS8_UK$VICT, JSS8_UK$ILOC, use =
"pairwise.complete.obs")[["estimate"]][["cor"]])
cor.test(JSS8_UK$OBSE, JSS8_UK$ILOC, use = "pairwise.complete.obs")
UK_OBSE_cor <- c(UK_OBSE_cor, cor.test(JSS8_UK$OBSE, JSS8_UK$ILOC, use =
"pairwise.complete.obs")[["estimate"]][["cor"]])
cor.test(JSS8_UK$BENE, JSS8_UK$ILOC, use = "pairwise.complete.obs")
UK_BENE_cor <- c(UK_BENE_cor, cor.test(JSS8_UK$BENE, JSS8_UK$ILOC, use =
"pairwise.complete.obs")[["estimate"]][["cor"]])
cor.test(JSS8_UK$OFFE, JSS8_UK$ILOC, use = "pairwise.complete.obs")
UK_OFFE_cor <- c(UK_OFFE_cor, cor.test(JSS8_UK$OFFE, JSS8_UK$ILOC, use =
"pairwise.complete.obs")[["estimate"]][["cor"]])

cor.test(JSS8_UK$VICT, JSS8_UK$ELOC, use = "pairwise.complete.obs")
UK_VICT_cor <- c(UK_VICT_cor, cor.test(JSS8_UK$VICT, JSS8_UK$ELOC, use =
"pairwise.complete.obs")[["estimate"]][["cor"]])
cor.test(JSS8_UK$OBSE, JSS8_UK$ELOC, use = "pairwise.complete.obs")
UK_OBSE_cor <- c(UK_OBSE_cor, cor.test(JSS8_UK$OBSE, JSS8_UK$ELOC, use =
"pairwise.complete.obs")[["estimate"]][["cor"]])
cor.test(JSS8_UK$BENE, JSS8_UK$ELOC, use = "pairwise.complete.obs")
UK_BENE_cor <- c(UK_BENE_cor, cor.test(JSS8_UK$BENE, JSS8_UK$ELOC, use =
"pairwise.complete.obs")[["estimate"]][["cor"]])
cor.test(JSS8_UK$OFFE, JSS8_UK$ELOC, use = "pairwise.complete.obs")
UK_OFFE_cor <- c(UK_OFFE_cor, cor.test(JSS8_UK$OFFE, JSS8_UK$ELOC, use =
"pairwise.complete.obs")[["estimate"]][["cor"]])

############################
#Interpersonal trust (KUSIV3)
############################
JSS8_UK$KUSIV <- (JSS8_UK$KUSI1+JSS8_UK$KUSI2R+JSS8_UK$KUSI3)/3

describe(JSS8_UK$KUSIV)
mvn(data = JSS8_UK[, c("KUSI1", "KUSI2R", "KUSI3")], mvnTest = "hz")$multivariateNormality
```

```
KUSIV_MM <- 'LV =~ KUSI1+KUSI2R+KUSI3

        KUSI1 ~ 0*1
        LV ~ NA*1'

KUSIV_MM.fit_UK <- sem(KUSIV_MM, data = JSS8_UK, estimator = "mlr", missing = "fiml",
std.lv = FALSE)
semTools::reliability(KUSIV_MM.fit_UK)

cor.test(JSS8_UK$VICT, JSS8_UK$KUSIV, use = "pairwise.complete.obs")
UK_VICT_cor <- c(UK_VICT_cor, cor.test(JSS8_UK$VICT, JSS8_UK$KUSIV, use =
"pairwise.complete.obs")[["estimate"]][["cor"]])
cor.test(JSS8_UK$OBSE, JSS8_UK$KUSIV, use = "pairwise.complete.obs")
UK_OBSE_cor <- c(UK_OBSE_cor, cor.test(JSS8_UK$OBSE, JSS8_UK$KUSIV, use =
"pairwise.complete.obs")[["estimate"]][["cor"]])
cor.test(JSS8_UK$BENE, JSS8_UK$KUSIV, use = "pairwise.complete.obs")
UK_BENE_cor <- c(UK_BENE_cor, cor.test(JSS8_UK$BENE, JSS8_UK$KUSIV, use =
"pairwise.complete.obs")[["estimate"]][["cor"]])
cor.test(JSS8_UK$OFFE, JSS8_UK$KUSIV, use = "pairwise.complete.obs")
UK_OFFE_cor <- c(UK_OFFE_cor, cor.test(JSS8_UK$OFFE, JSS8_UK$KUSIV, use =
"pairwise.complete.obs")[["estimate"]][["cor"]])

#################
#Optimism (SOP2)
#################
JSS8_UK$SOP <- (JSS8_UK$PESS1R + JSS8_UK$OPTI1)/2

describe(JSS8_UK$SOP)
mvn(data = JSS8_UK[, c("PESS1R", "OPTI1")], mvnTest = "hz")$multivariateNormality


SOP_MM <- 'LV =~ c(a)*PESS1R+c(a)*OPTI1

        PESS1R ~ 0*1
        LV ~ NA*1'

SOP_MM.fit_UK <- sem(SOP_MM, data = JSS8_UK, estimator = "mlr", missing = "fiml", std.lv =
FALSE)
semTools::reliability(SOP_MM.fit_UK)

cor.test(JSS8_UK$VICT, JSS8_UK$SOP, use = "pairwise.complete.obs")
UK_VICT_cor <- c(UK_VICT_cor, cor.test(JSS8_UK$VICT, JSS8_UK$SOP, use =
"pairwise.complete.obs")[["estimate"]][["cor"]])
cor.test(JSS8_UK$OBSE, JSS8_UK$SOP, use = "pairwise.complete.obs")
UK_OBSE_cor <- c(UK_OBSE_cor, cor.test(JSS8_UK$OBSE, JSS8_UK$SOP, use =
"pairwise.complete.obs")[["estimate"]][["cor"]])
cor.test(JSS8_UK$BENE, JSS8_UK$SOP, use = "pairwise.complete.obs")
UK_BENE_cor <- c(UK_BENE_cor, cor.test(JSS8_UK$BENE, JSS8_UK$SOP, use =
"pairwise.complete.obs")[["estimate"]][["cor"]])
cor.test(JSS8_UK$OFFE, JSS8_UK$SOP, use = "pairwise.complete.obs")
UK_OFFE_cor <- c(UK_OFFE_cor, cor.test(JSS8_UK$OFFE, JSS8_UK$SOP, use =
"pairwise.complete.obs")[["estimate"]][["cor"]])

################################
```

```
#General life satisfaction (L-1)
##################################
describe(JSS8_UK$LISA1)

cor.test(JSS8_UK$VICT, JSS8_UK$LISA1, use = "pairwise.complete.obs")
UK_VICT_cor <- c(UK_VICT_cor, cor.test(JSS8_UK$VICT, JSS8_UK$LISA1, use =
"pairwise.complete.obs")[["estimate"]][["cor"]])
cor.test(JSS8_UK$OBSE, JSS8_UK$LISA1, use = "pairwise.complete.obs")
UK_OBSE_cor <- c(UK_OBSE_cor, cor.test(JSS8_UK$OBSE, JSS8_UK$LISA1, use =
"pairwise.complete.obs")[["estimate"]][["cor"]])
cor.test(JSS8_UK$BENE, JSS8_UK$LISA1, use = "pairwise.complete.obs")
UK_BENE_cor <- c(UK_BENE_cor, cor.test(JSS8_UK$BENE, JSS8_UK$LISA1, use =
"pairwise.complete.obs")[["estimate"]][["cor"]])
cor.test(JSS8_UK$OFFE, JSS8_UK$LISA1, use = "pairwise.complete.obs")
UK_OFFE_cor <- c(UK_OFFE_cor, cor.test(JSS8_UK$OFFE, JSS8_UK$LISA1, use =
"pairwise.complete.obs")[["estimate"]][["cor"]])

############################
#Left-Right self-placement
############################
describe(JSS8_UK$LRPL1)

cor.test(JSS8_UK$VICT, JSS8_UK$LRPL1, use = "pairwise.complete.obs")
UK_VICT_cor <- c(UK_VICT_cor, cor.test(JSS8_UK$VICT, JSS8_UK$LRPL1, use =
"pairwise.complete.obs")[["estimate"]][["cor"]])
cor.test(JSS8_UK$OBSE, JSS8_UK$LRPL1, use = "pairwise.complete.obs")
UK_OBSE_cor <- c(UK_OBSE_cor, cor.test(JSS8_UK$OBSE, JSS8_UK$LRPL1, use =
"pairwise.complete.obs")[["estimate"]][["cor"]])
cor.test(JSS8_UK$BENE, JSS8_UK$LRPL1, use = "pairwise.complete.obs")
UK_BENE_cor <- c(UK_BENE_cor, cor.test(JSS8_UK$BENE, JSS8_UK$LRPL1, use =
"pairwise.complete.obs")[["estimate"]][["cor"]])
cor.test(JSS8_UK$OFFE, JSS8_UK$LRPL1, use = "pairwise.complete.obs")
UK_OFFE_cor <- c(UK_OFFE_cor, cor.test(JSS8_UK$OFFE, JSS8_UK$LRPL1, use =
"pairwise.complete.obs")[["estimate"]][["cor"]])

###########################
#Authoritarianism (KSA-3)
###########################
JSS8_UK$AAGG <-
(JSS8_UK$AAGG1+JSS8_UK$AAGG2+JSS8_UK$AAGG3+JSS8_UK$ASUB1)/4
JSS8_UK$ASUB <- (JSS8_UK$ASUB2+JSS8_UK$ASUB3)/2
JSS8_UK$CONV <- (JSS8_UK$CONV1+JSS8_UK$CONV2+JSS8_UK$CONV3)/3

describe(JSS8_UK$AAGG)
mvn(data = JSS8_UK[, c("AAGG1", "AAGG2", "AAGG3", "ASUB1")], mvnTest =
"hz")$multivariateNormality
describe(JSS8_UK$ASUB)
mvn(data = JSS8_UK[, c("ASUB2", "ASUB3")], mvnTest = "hz")$multivariateNormality
describe(JSS8_UK$CONV)
mvn(data = JSS8_UK[, c("CONV1", "CONV2", "CONV3")], mvnTest = "hz")$multivariateNormality

KSA_MM <- 'LV_AAGG =~ AAGG1+AAGG2+AAGG3+ASUB1
        LV_ASUB =~ c(a)*ASUB2+c(a)*ASUB3
        LV_CONV =~ CONV1+CONV2+CONV3
```

```
        AAGG1+ASUB2+CONV1 ~ 0*1
        LV_AAGG+LV_ASUB+LV_CONV ~ NA*1'

KSA_MM.fit_UK <- sem(KSA_MM, data = JSS8_UK, estimator = "mlr", missing = "fiml", std.lv =
FALSE)
semTools::reliability(KSA_MM.fit_UK)

cor.test(JSS8_UK$VICT, JSS8_UK$AAGG, use = "pairwise.complete.obs")
UK_VICT_cor <- c(UK_VICT_cor, cor.test(JSS8_UK$VICT, JSS8_UK$AAGG, use =
"pairwise.complete.obs")[["estimate"]][["cor"]])
cor.test(JSS8_UK$OBSE, JSS8_UK$AAGG, use = "pairwise.complete.obs")
UK_OBSE_cor <- c(UK_OBSE_cor, cor.test(JSS8_UK$OBSE, JSS8_UK$AAGG, use =
"pairwise.complete.obs")[["estimate"]][["cor"]])
cor.test(JSS8_UK$BENE, JSS8_UK$AAGG, use = "pairwise.complete.obs")
UK_BENE_cor <- c(UK_BENE_cor, cor.test(JSS8_UK$BENE, JSS8_UK$AAGG, use =
"pairwise.complete.obs")[["estimate"]][["cor"]])
cor.test(JSS8_UK$OFFE, JSS8_UK$AAGG, use = "pairwise.complete.obs")
UK_OFFE_cor <- c(UK_OFFE_cor, cor.test(JSS8_UK$OFFE, JSS8_UK$AAGG, use =
"pairwise.complete.obs")[["estimate"]][["cor"]])

cor.test(JSS8_UK$VICT, JSS8_UK$ASUB, use = "pairwise.complete.obs")
UK_VICT_cor <- c(UK_VICT_cor, cor.test(JSS8_UK$VICT, JSS8_UK$ASUB, use =
"pairwise.complete.obs")[["estimate"]][["cor"]])
cor.test(JSS8_UK$OBSE, JSS8_UK$ASUB, use = "pairwise.complete.obs")
UK_OBSE_cor <- c(UK_OBSE_cor, cor.test(JSS8_UK$OBSE, JSS8_UK$ASUB, use =
"pairwise.complete.obs")[["estimate"]][["cor"]])
cor.test(JSS8_UK$BENE, JSS8_UK$ASUB, use = "pairwise.complete.obs")
UK_BENE_cor <- c(UK_BENE_cor, cor.test(JSS8_UK$BENE, JSS8_UK$ASUB, use =
"pairwise.complete.obs")[["estimate"]][["cor"]])
cor.test(JSS8_UK$OFFE, JSS8_UK$ASUB, use = "pairwise.complete.obs")
UK_OFFE_cor <- c(UK_OFFE_cor, cor.test(JSS8_UK$OFFE, JSS8_UK$ASUB, use =
"pairwise.complete.obs")[["estimate"]][["cor"]])

cor.test(JSS8_UK$VICT, JSS8_UK$CONV, use = "pairwise.complete.obs")
UK_VICT_cor <- c(UK_VICT_cor, cor.test(JSS8_UK$VICT, JSS8_UK$CONV, use =
"pairwise.complete.obs")[["estimate"]][["cor"]])
cor.test(JSS8_UK$OBSE, JSS8_UK$CONV, use = "pairwise.complete.obs")
UK_OBSE_cor <- c(UK_OBSE_cor, cor.test(JSS8_UK$OBSE, JSS8_UK$CONV, use =
"pairwise.complete.obs")[["estimate"]][["cor"]])
cor.test(JSS8_UK$BENE, JSS8_UK$CONV, use = "pairwise.complete.obs")
UK_BENE_cor <- c(UK_BENE_cor, cor.test(JSS8_UK$BENE, JSS8_UK$CONV, use =
"pairwise.complete.obs")[["estimate"]][["cor"]])
cor.test(JSS8_UK$OFFE, JSS8_UK$CONV, use = "pairwise.complete.obs")
UK_OFFE_cor <- c(UK_OFFE_cor, cor.test(JSS8_UK$OFFE, JSS8_UK$CONV, use =
"pairwise.complete.obs")[["estimate"]][["cor"]])

##############################
#Social desirability (KSE-G)
##############################
JSS8_UK$SDPQ <- (JSS8_UK$SDPQ1+JSS8_UK$SDPQ2+JSS8_UK$SDPQ3)/3
JSS8_UK$SDNQ <- (JSS8_UK$SDNQ1+JSS8_UK$SDNQ2+JSS8_UK$SDNQ3)/3

describe(JSS8_UK$SDPQ)
```

```
describe(JSS8_UK$SDNQ)
mvn(data = JSS8_UK[, c("SDPQ1", "SDPQ2", "SDPQ3")], mvnTest = "hz")$multivariateNormality
mvn(data = JSS8_UK[, c("SDNQ1", "SDNQ2", "SDNQ3")], mvnTest = "hz")$multivariateNormality

KSEG_MM <- 'LV_SDPQ =~ SDPQ1+SDPQ2+SDPQ3
	LV_SDNQ =~ SDNQ1+SDNQ2+SDNQ3

	SDPQ1+SDNQ1 ~ 0*1
	LV_SDPQ+LV_SDNQ ~ NA*1'

KSEG_MM.fit_UK <- sem(KSEG_MM, data = JSS8_UK, estimator = "mlr", missing = "fiml", std.lv
= FALSE)
semTools::reliability(KSEG_MM.fit_UK)

cor.test(JSS8_UK$VICT, JSS8_UK$SDPQ, use = "pairwise.complete.obs")
UK_VICT_cor <- c(UK_VICT_cor, cor.test(JSS8_UK$VICT, JSS8_UK$SDPQ, use =
"pairwise.complete.obs")[["estimate"]][["cor"]])
cor.test(JSS8_UK$OBSE, JSS8_UK$SDPQ, use = "pairwise.complete.obs")
UK_OBSE_cor <- c(UK_OBSE_cor, cor.test(JSS8_UK$OBSE, JSS8_UK$SDPQ, use =
"pairwise.complete.obs")[["estimate"]][["cor"]])
cor.test(JSS8_UK$BENE, JSS8_UK$SDPQ, use = "pairwise.complete.obs")
UK_BENE_cor <- c(UK_BENE_cor, cor.test(JSS8_UK$BENE, JSS8_UK$SDPQ, use =
"pairwise.complete.obs")[["estimate"]][["cor"]])
cor.test(JSS8_UK$OFFE, JSS8_UK$SDPQ, use = "pairwise.complete.obs")
UK_OFFE_cor <- c(UK_OFFE_cor, cor.test(JSS8_UK$OFFE, JSS8_UK$SDPQ, use =
"pairwise.complete.obs")[["estimate"]][["cor"]])

cor.test(JSS8_UK$VICT, JSS8_UK$SDNQ, use = "pairwise.complete.obs")
UK_VICT_cor <- c(UK_VICT_cor, cor.test(JSS8_UK$VICT, JSS8_UK$SDNQ, use =
"pairwise.complete.obs")[["estimate"]][["cor"]])
cor.test(JSS8_UK$OBSE, JSS8_UK$SDNQ, use = "pairwise.complete.obs")
UK_OBSE_cor <- c(UK_OBSE_cor, cor.test(JSS8_UK$OBSE, JSS8_UK$SDNQ, use =
"pairwise.complete.obs")[["estimate"]][["cor"]])
cor.test(JSS8_UK$BENE, JSS8_UK$SDNQ, use = "pairwise.complete.obs")
UK_BENE_cor <- c(UK_BENE_cor, cor.test(JSS8_UK$BENE, JSS8_UK$SDNQ, use =
"pairwise.complete.obs")[["estimate"]][["cor"]])
cor.test(JSS8_UK$OFFE, JSS8_UK$SDNQ, use = "pairwise.complete.obs")
UK_OFFE_cor <- c(UK_OFFE_cor, cor.test(JSS8_UK$OFFE, JSS8_UK$SDNQ, use =
"pairwise.complete.obs")[["estimate"]][["cor"]])

########
#Health
########
describe(JSS8_UK$HEAL)

cor.test(JSS8_UK$VICT, JSS8_UK$HEAL, use = "pairwise.complete.obs")
cor.test(JSS8_UK$VICT, JSS8_UK$HEAL, use = "pairwise.complete.obs")$p.value # p-value below
0.05?
UK_VICT_cor <- c(UK_VICT_cor, cor.test(JSS8_UK$VICT, JSS8_UK$HEAL, use =
"pairwise.complete.obs")[["estimate"]][["cor"]])
cor.test(JSS8_UK$OBSE, JSS8_UK$HEAL, use = "pairwise.complete.obs")
UK_OBSE_cor <- c(UK_OBSE_cor, cor.test(JSS8_UK$OBSE, JSS8_UK$HEAL, use =
"pairwise.complete.obs")[["estimate"]][["cor"]])
cor.test(JSS8_UK$BENE, JSS8_UK$HEAL, use = "pairwise.complete.obs")
```

```
UK_BENE_cor <- c(UK_BENE_cor, cor.test(JSS8_UK$BENE, JSS8_UK$HEAL, use =
"pairwise.complete.obs")[["estimate"]][["cor"]])
cor.test(JSS8_UK$OFFE, JSS8_UK$HEAL, use = "pairwise.complete.obs")
cor.test(JSS8_UK$OFFE, JSS8_UK$HEAL, use = "pairwise.complete.obs")$p.value # p-value below
0.05?
UK_OFFE_cor <- c(UK_OFFE_cor, cor.test(JSS8_UK$OFFE, JSS8_UK$HEAL, use =
"pairwise.complete.obs")[["estimate"]][["cor"]])

###################
#Employment status
###################
#1) employed
#2) self-employed
#3) out of work and looking for work
#4) out of work but not currently looking for work
#5) doing housework
#6) pupil/student
#7) apprentice/internship
#8) retired
#[9) none of what is mentioned above]
describe(JSS8_UK$EMPL)
#unemployed vs. employed
JSS8_UK$EMPL.new <- recode(JSS8_UK$EMPL, "1:2 = 2; 3:4 = 1; else = NA")
describe(JSS8_UK$EMPL.new)

cor.test(JSS8_UK$VICT, JSS8_UK$EMPL.new, use = "pairwise.complete.obs")
UK_VICT_cor <- c(UK_VICT_cor, cor.test(JSS8_UK$VICT, JSS8_UK$EMPL.new, use =
"pairwise.complete.obs")[["estimate"]][["cor"]])
cor.test(JSS8_UK$OBSE, JSS8_UK$EMPL.new, use = "pairwise.complete.obs")
UK_OBSE_cor <- c(UK_OBSE_cor, cor.test(JSS8_UK$OBSE, JSS8_UK$EMPL.new, use =
"pairwise.complete.obs")[["estimate"]][["cor"]])
cor.test(JSS8_UK$BENE, JSS8_UK$EMPL.new, use = "pairwise.complete.obs")
UK_BENE_cor <- c(UK_BENE_cor, cor.test(JSS8_UK$BENE, JSS8_UK$EMPL.new, use =
"pairwise.complete.obs")[["estimate"]][["cor"]])
cor.test(JSS8_UK$OFFE, JSS8_UK$EMPL.new, use = "pairwise.complete.obs")
UK_OFFE_cor <- c(UK_OFFE_cor, cor.test(JSS8_UK$OFFE, JSS8_UK$EMPL.new, use =
"pairwise.complete.obs")[["estimate"]][["cor"]])

########
#Income
########
describe(JSS8_UK$INCO)

cor.test(JSS8_UK$VICT, JSS8_UK$INCO, use = "pairwise.complete.obs")
UK_VICT_cor <- c(UK_VICT_cor, cor.test(JSS8_UK$VICT, JSS8_UK$INCO, use =
"pairwise.complete.obs")[["estimate"]][["cor"]])
cor.test(JSS8_UK$OBSE, JSS8_UK$INCO, use = "pairwise.complete.obs")
UK_OBSE_cor <- c(UK_OBSE_cor, cor.test(JSS8_UK$OBSE, JSS8_UK$INCO, use =
"pairwise.complete.obs")[["estimate"]][["cor"]])
cor.test(JSS8_UK$BENE, JSS8_UK$INCO, use = "pairwise.complete.obs")
UK_BENE_cor <- c(UK_BENE_cor, cor.test(JSS8_UK$BENE, JSS8_UK$INCO, use =
"pairwise.complete.obs")[["estimate"]][["cor"]])
cor.test(JSS8_UK$OFFE, JSS8_UK$INCO, use = "pairwise.complete.obs")
```

```
UK_OFFE_cor <- c(UK_OFFE_cor, cor.test(JSS8_UK$OFFE, JSS8_UK$INCO, use =
"pairwise.complete.obs")[["estimate"]][["cor"]])

########################
#Educational attainment
########################
describe(JSS8_UK$SCHO)

cor.test(JSS8_UK$VICT, JSS8_UK$SCHO, use = "pairwise.complete.obs")
UK_VICT_cor <- c(UK_VICT_cor, cor.test(JSS8_UK$VICT, JSS8_UK$SCHO, use =
"pairwise.complete.obs")[["estimate"]][["cor"]])
cor.test(JSS8_UK$OBSE, JSS8_UK$SCHO, use = "pairwise.complete.obs")
UK_OBSE_cor <- c(UK_OBSE_cor, cor.test(JSS8_UK$OBSE, JSS8_UK$SCHO, use =
"pairwise.complete.obs")[["estimate"]][["cor"]])
cor.test(JSS8_UK$BENE, JSS8_UK$SCHO, use = "pairwise.complete.obs")
UK_BENE_cor <- c(UK_BENE_cor, cor.test(JSS8_UK$BENE, JSS8_UK$SCHO, use =
"pairwise.complete.obs")[["estimate"]][["cor"]])
cor.test(JSS8_UK$OFFE, JSS8_UK$SCHO, use = "pairwise.complete.obs")
UK_OFFE_cor <- c(UK_OFFE_cor, cor.test(JSS8_UK$OFFE, JSS8_UK$SCHO, use =
"pairwise.complete.obs")[["estimate"]][["cor"]])

#####
#Age
#####
describe(JSS8_UK$AGE)

cor.test(JSS8_UK$VICT, JSS8_UK$AGE, use = "pairwise.complete.obs")
UK_VICT_cor <- c(UK_VICT_cor, cor.test(JSS8_UK$VICT, JSS8_UK$AGE, use =
"pairwise.complete.obs")[["estimate"]][["cor"]])
cor.test(JSS8_UK$OBSE, JSS8_UK$AGE, use = "pairwise.complete.obs")
UK_OBSE_cor <- c(UK_OBSE_cor, cor.test(JSS8_UK$OBSE, JSS8_UK$AGE, use =
"pairwise.complete.obs")[["estimate"]][["cor"]])
cor.test(JSS8_UK$BENE, JSS8_UK$AGE, use = "pairwise.complete.obs")
UK_BENE_cor <- c(UK_BENE_cor, cor.test(JSS8_UK$BENE, JSS8_UK$AGE, use =
"pairwise.complete.obs")[["estimate"]][["cor"]])
cor.test(JSS8_UK$OFFE, JSS8_UK$AGE, use = "pairwise.complete.obs")
UK_OFFE_cor <- c(UK_OFFE_cor, cor.test(JSS8_UK$OFFE, JSS8_UK$AGE, use =
"pairwise.complete.obs")[["estimate"]][["cor"]])

########
#Gender
########
describe(JSS8_UK$SEX)

cor.test(JSS8_UK$VICT, JSS8_UK$SEX, use = "pairwise.complete.obs")
UK_VICT_cor <- c(UK_VICT_cor, cor.test(JSS8_UK$VICT, JSS8_UK$SEX, use =
"pairwise.complete.obs")[["estimate"]][["cor"]])
cor.test(JSS8_UK$OBSE, JSS8_UK$SEX, use = "pairwise.complete.obs")
UK_OBSE_cor <- c(UK_OBSE_cor, cor.test(JSS8_UK$OBSE, JSS8_UK$SEX, use =
"pairwise.complete.obs")[["estimate"]][["cor"]])
cor.test(JSS8_UK$BENE, JSS8_UK$SEX, use = "pairwise.complete.obs")
UK_BENE_cor <- c(UK_BENE_cor, cor.test(JSS8_UK$BENE, JSS8_UK$SEX, use =
"pairwise.complete.obs")[["estimate"]][["cor"]])
cor.test(JSS8_UK$OFFE, JSS8_UK$SEX, use = "pairwise.complete.obs")
```

```
UK_OFFE_cor <- c(UK_OFFE_cor, cor.test(JSS8_UK$OFFE, JSS8_UK$SEX, use =
"pairwise.complete.obs")[["estimate"]][["cor"]])

##########################################################################

##Germany

#Empty vectors to save correlations
D_VICT_cor <- c()
D_OBSE_cor <- c()
D_BENE_cor <- c()
D_OFFE_cor <- c()

dimension.matrix <- list(c("EXTR", "AGRE", "CONS", "NEGA", "OPEN", "ASKU", "ILOC",
"ELOC",
                     "KUSIV", "SOP", "LISA1", "LRPL1", "AAGG", "ASUB", "CONV",
                     "SDPQ", "SDNQ", "HEAL", "EMPL.new", "INCO", "SCHO", "AGE", "SEX"),
                   c("UK", "D"))

##############################
#Justice sensitivity (USS-8)
#############################
JSS8_D$VICT <- (JSS8_D$VICT1+JSS8_D$VICT2)/2
JSS8_D$OBSE <- (JSS8_D$OBSE1+JSS8_D$OBSE2)/2
JSS8_D$BENE <- (JSS8_D$BENE1+JSS8_D$BENE2)/2
JSS8_D$OFFE <- (JSS8_D$OFFE1+JSS8_D$OFFE2)/2

#####################
#Big Five (BFI-2-XS)
#####################
JSS8_D$EXTR <- (JSS8_D$EXTR1R+JSS8_D$EXTR2+JSS8_D$EXTR3)/3 #Extraversion
JSS8_D$AGRE <- (JSS8_D$AGRE1+JSS8_D$AGRE2R+JSS8_D$AGRE3)/3 #Agreeableness
JSS8_D$CONS <- (JSS8_D$CONS1R+JSS8_D$CONS2R+JSS8_D$CONS3)/3 #Conscientiousness
JSS8_D$NEGA <- (JSS8_D$NEGA1+JSS8_D$NEGA2+JSS8_D$NEGA3R)/3 #Neuroticism
JSS8_D$OPEN <- (JSS8_D$OPEN1+JSS8_D$OPEN2R+JSS8_D$OPEN3)/3 #Openness

describe(JSS8_D$EXTR)
mvn(data = JSS8_D[, c("EXTR1R", "EXTR2", "EXTR3")], mvnTest = "hz")$multivariateNormality
describe(JSS8_D$AGRE)
mvn(data = JSS8_D[, c("AGRE1", "AGRE2R", "AGRE3")], mvnTest = "hz")$multivariateNormality
describe(JSS8_D$CONS)
mvn(data = JSS8_D[, c("CONS1R", "CONS2R", "CONS3")], mvnTest = "hz")$multivariateNormality
describe(JSS8_D$NEGA)
mvn(data = JSS8_D[, c("NEGA1", "NEGA2", "NEGA3R")], mvnTest = "hz")$multivariateNormality
describe(JSS8_D$OPEN)
mvn(data = JSS8_D[, c("OPEN1", "OPEN2R", "OPEN3")], mvnTest = "hz")$multivariateNormality

BFI_MM <- 'LV_EXTR =~ EXTR1R+EXTR2+EXTR3
      LV_AGRE =~ AGRE1+AGRE2R+AGRE3
      LV_CONS =~ CONS1R+CONS2R+CONS3
      LV_NEGA =~ NEGA1+NEGA2+NEGA3R
      LV_OPEN =~ OPEN1+OPEN2R+OPEN3

      EXTR1R+AGRE1+CONS1R+NEGA1+OPEN1 ~ 0*1
```

```
    LV_EXTR+LV_AGRE+LV_CONS+LV_NEGA+LV_OPEN ~ NA*1

    AGRE2R ~~ c(a1)*AGRE2R
    a1 == 0'

BFI_MM.fit_D <- sem(BFI_MM, data = JSS8_D, estimator = "mlr", missing = "fiml", std.lv =
FALSE)
semTools::reliability(BFI_MM.fit_D)

cor.test(JSS8_D$VICT, JSS8_D$EXTR, use = "pairwise.complete.obs")
D_VICT_cor <- c(D_VICT_cor, cor.test(JSS8_D$VICT, JSS8_D$EXTR, use =
"pairwise.complete.obs")[["estimate"]][["cor"]])
cor.test(JSS8_D$OBSE, JSS8_D$EXTR, use = "pairwise.complete.obs")
D_OBSE_cor <- c(D_OBSE_cor, cor.test(JSS8_D$OBSE, JSS8_D$EXTR, use =
"pairwise.complete.obs")[["estimate"]][["cor"]])
cor.test(JSS8_D$BENE, JSS8_D$EXTR, use = "pairwise.complete.obs")
D_BENE_cor <- c(D_BENE_cor, cor.test(JSS8_D$BENE, JSS8_D$EXTR, use =
"pairwise.complete.obs")[["estimate"]][["cor"]])
cor.test(JSS8_D$OFFE, JSS8_D$EXTR, use = "pairwise.complete.obs")
D_OFFE_cor <- c(D_OFFE_cor, cor.test(JSS8_D$OFFE, JSS8_D$EXTR, use =
"pairwise.complete.obs")[["estimate"]][["cor"]])

cor.test(JSS8_D$VICT, JSS8_D$AGRE, use = "pairwise.complete.obs")
D_VICT_cor <- c(D_VICT_cor, cor.test(JSS8_D$VICT, JSS8_D$AGRE, use =
"pairwise.complete.obs")[["estimate"]][["cor"]])
cor.test(JSS8_D$OBSE, JSS8_D$AGRE, use = "pairwise.complete.obs")
D_OBSE_cor <- c(D_OBSE_cor, cor.test(JSS8_D$OBSE, JSS8_D$AGRE, use =
"pairwise.complete.obs")[["estimate"]][["cor"]])
cor.test(JSS8_D$BENE, JSS8_D$AGRE, use = "pairwise.complete.obs")
D_BENE_cor <- c(D_BENE_cor, cor.test(JSS8_D$BENE, JSS8_D$AGRE, use =
"pairwise.complete.obs")[["estimate"]][["cor"]])
cor.test(JSS8_D$OFFE, JSS8_D$AGRE, use = "pairwise.complete.obs")
D_OFFE_cor <- c(D_OFFE_cor, cor.test(JSS8_D$OFFE, JSS8_D$AGRE, use =
"pairwise.complete.obs")[["estimate"]][["cor"]])

cor.test(JSS8_D$VICT, JSS8_D$CONS, use = "pairwise.complete.obs")
D_VICT_cor <- c(D_VICT_cor, cor.test(JSS8_D$VICT, JSS8_D$CONS, use =
"pairwise.complete.obs")[["estimate"]][["cor"]])
cor.test(JSS8_D$OBSE, JSS8_D$CONS, use = "pairwise.complete.obs")
D_OBSE_cor <- c(D_OBSE_cor, cor.test(JSS8_D$OBSE, JSS8_D$CONS, use =
"pairwise.complete.obs")[["estimate"]][["cor"]])
cor.test(JSS8_D$BENE, JSS8_D$CONS, use = "pairwise.complete.obs")
D_BENE_cor <- c(D_BENE_cor, cor.test(JSS8_D$BENE, JSS8_D$CONS, use =
"pairwise.complete.obs")[["estimate"]][["cor"]])
cor.test(JSS8_D$OFFE, JSS8_D$CONS, use = "pairwise.complete.obs")
D_OFFE_cor <- c(D_OFFE_cor, cor.test(JSS8_D$OFFE, JSS8_D$CONS, use =
"pairwise.complete.obs")[["estimate"]][["cor"]])

cor.test(JSS8_D$VICT, JSS8_D$NEGA, use = "pairwise.complete.obs")
D_VICT_cor <- c(D_VICT_cor, cor.test(JSS8_D$VICT, JSS8_D$NEGA, use =
"pairwise.complete.obs")[["estimate"]][["cor"]])
cor.test(JSS8_D$OBSE, JSS8_D$NEGA, use = "pairwise.complete.obs")
D_OBSE_cor <- c(D_OBSE_cor, cor.test(JSS8_D$OBSE, JSS8_D$NEGA, use =
"pairwise.complete.obs")[["estimate"]][["cor"]])
```

```
cor.test(JSS8_D$BENE, JSS8_D$NEGA, use = "pairwise.complete.obs")
D_BENE_cor <- c(D_BENE_cor, cor.test(JSS8_D$BENE, JSS8_D$NEGA, use =
"pairwise.complete.obs")[["estimate"]][["cor"]])
cor.test(JSS8_D$OFFE, JSS8_D$NEGA, use = "pairwise.complete.obs")
D_OFFE_cor <- c(D_OFFE_cor, cor.test(JSS8_D$OFFE, JSS8_D$NEGA, use =
"pairwise.complete.obs")[["estimate"]][["cor"]])

cor.test(JSS8_D$VICT, JSS8_D$OPEN, use = "pairwise.complete.obs")
D_VICT_cor <- c(D_VICT_cor, cor.test(JSS8_D$VICT, JSS8_D$OPEN, use =
"pairwise.complete.obs")[["estimate"]][["cor"]])
cor.test(JSS8_D$OBSE, JSS8_D$OPEN, use = "pairwise.complete.obs")
D_OBSE_cor <- c(D_OBSE_cor, cor.test(JSS8_D$OBSE, JSS8_D$OPEN, use =
"pairwise.complete.obs")[["estimate"]][["cor"]])
cor.test(JSS8_D$BENE, JSS8_D$OPEN, use = "pairwise.complete.obs")
D_BENE_cor <- c(D_BENE_cor, cor.test(JSS8_D$BENE, JSS8_D$OPEN, use =
"pairwise.complete.obs")[["estimate"]][["cor"]])
cor.test(JSS8_D$OFFE, JSS8_D$OPEN, use = "pairwise.complete.obs")
D_OFFE_cor <- c(D_OFFE_cor, cor.test(JSS8_D$OFFE, JSS8_D$OPEN, use =
"pairwise.complete.obs")[["estimate"]][["cor"]])


################################
#General self-efficacy (ASKU)
################################
JSS8_D$ASKU <- (JSS8_D$ASKU1+JSS8_D$ASKU2+JSS8_D$ASKU3)/3

describe(JSS8_D$ASKU)
mvn(data = JSS8_D[, c("ASKU1", "ASKU2", "ASKU3")], mvnTest = "hz")$multivariateNormality


ASKU_MM <- 'LV =~ ASKU1+ASKU2+ASKU3

	ASKU1 ~ 0*1
	LV ~ NA*1'

ASKU_MM.fit_D <- sem(ASKU_MM, data = JSS8_D, estimator = "mlr", missing = "fiml", std.lv =
FALSE)
semTools::reliability(ASKU_MM.fit_D)

cor.test(JSS8_D$VICT, JSS8_D$ASKU, use = "pairwise.complete.obs")
D_VICT_cor <- c(D_VICT_cor, cor.test(JSS8_D$VICT, JSS8_D$ASKU, use =
"pairwise.complete.obs")[["estimate"]][["cor"]])
cor.test(JSS8_D$OBSE, JSS8_D$ASKU, use = "pairwise.complete.obs")
D_OBSE_cor <- c(D_OBSE_cor, cor.test(JSS8_D$OBSE, JSS8_D$ASKU, use =
"pairwise.complete.obs")[["estimate"]][["cor"]])
cor.test(JSS8_D$BENE, JSS8_D$ASKU, use = "pairwise.complete.obs")
D_BENE_cor <- c(D_BENE_cor, cor.test(JSS8_D$BENE, JSS8_D$ASKU, use =
"pairwise.complete.obs")[["estimate"]][["cor"]])
cor.test(JSS8_D$OFFE, JSS8_D$ASKU, use = "pairwise.complete.obs")
D_OFFE_cor <- c(D_OFFE_cor, cor.test(JSS8_D$OFFE, JSS8_D$ASKU, use =
"pairwise.complete.obs")[["estimate"]][["cor"]])

##########################################
#Internal-external locus of control (IE-4)
```

```
########################################
JSS8_D$ILOC <- (JSS8_D$ILOC1+JSS8_D$ILOC2)/2
JSS8_D$ELOC <- (JSS8_D$ELOC1+JSS8_D$ELOC2)/2

describe(JSS8_D$ILOC)
describe(JSS8_D$ELOC)
mvn(data = JSS8_D[, c("ILOC1", "ILOC2")], mvnTest = "hz")$multivariateNormality
mvn(data = JSS8_D[, c("ELOC1", "ELOC2")], mvnTest = "hz")$multivariateNormality

LOC_MM <- 'LV_ILOC =~ c(a)*ILOC1+c(a)*ILOC2
	LV_ELOC =~ c(b)*ELOC1+c(b)*ELOC2

	ILOC1+ELOC1 ~ 0*1
	LV_ILOC+LV_ELOC ~ NA*1'

LOC_MM.fit_D <- sem(LOC_MM, data = JSS8_D, estimator = "mlr", missing = "fiml", std.lv =
FALSE)
semTools::reliability(LOC_MM.fit_D)

cor.test(JSS8_D$VICT, JSS8_D$ILOC, use = "pairwise.complete.obs")
D_VICT_cor <- c(D_VICT_cor, cor.test(JSS8_D$VICT, JSS8_D$ILOC, use =
"pairwise.complete.obs")[["estimate"]][["cor"]])
cor.test(JSS8_D$OBSE, JSS8_D$ILOC, use = "pairwise.complete.obs")
D_OBSE_cor <- c(D_OBSE_cor, cor.test(JSS8_D$OBSE, JSS8_D$ILOC, use =
"pairwise.complete.obs")[["estimate"]][["cor"]])
cor.test(JSS8_D$BENE, JSS8_D$ILOC, use = "pairwise.complete.obs")
D_BENE_cor <- c(D_BENE_cor, cor.test(JSS8_D$BENE, JSS8_D$ILOC, use =
"pairwise.complete.obs")[["estimate"]][["cor"]])
cor.test(JSS8_D$OFFE, JSS8_D$ILOC, use = "pairwise.complete.obs")
D_OFFE_cor <- c(D_OFFE_cor, cor.test(JSS8_D$OFFE, JSS8_D$ILOC, use =
"pairwise.complete.obs")[["estimate"]][["cor"]])

cor.test(JSS8_D$VICT, JSS8_D$ELOC, use = "pairwise.complete.obs")
D_VICT_cor <- c(D_VICT_cor, cor.test(JSS8_D$VICT, JSS8_D$ELOC, use =
"pairwise.complete.obs")[["estimate"]][["cor"]])
cor.test(JSS8_D$OBSE, JSS8_D$ELOC, use = "pairwise.complete.obs")
D_OBSE_cor <- c(D_OBSE_cor, cor.test(JSS8_D$OBSE, JSS8_D$ELOC, use =
"pairwise.complete.obs")[["estimate"]][["cor"]])
cor.test(JSS8_D$BENE, JSS8_D$ELOC, use = "pairwise.complete.obs")
D_BENE_cor <- c(D_BENE_cor, cor.test(JSS8_D$BENE, JSS8_D$ELOC, use =
"pairwise.complete.obs")[["estimate"]][["cor"]])
cor.test(JSS8_D$OFFE, JSS8_D$ELOC, use = "pairwise.complete.obs")
D_OFFE_cor <- c(D_OFFE_cor, cor.test(JSS8_D$OFFE, JSS8_D$ELOC, use =
"pairwise.complete.obs")[["estimate"]][["cor"]])

#############################
#Interpersonal trust (KUSIV3)
#############################
JSS8_D$KUSIV <- (JSS8_D$KUSI1+JSS8_D$KUSI2R+JSS8_D$KUSI3)/3

describe(JSS8_D$KUSIV)
mvn(data = JSS8_D[, c("KUSI1", "KUSI2R", "KUSI3")], mvnTest = "hz")$multivariateNormality
```

```
KUSIV_MM <- 'LV =~ KUSI1+KUSI2R+KUSI3

        KUSI1 ~ 0*1
        LV ~ NA*1'

KUSIV_MM.fit_D <- sem(KUSIV_MM, data = JSS8_D, estimator = "mlr", missing = "fiml", std.lv =
FALSE)
semTools::reliability(KUSIV_MM.fit_D)

cor.test(JSS8_D$VICT, JSS8_D$KUSIV, use = "pairwise.complete.obs")
D_VICT_cor <- c(D_VICT_cor, cor.test(JSS8_D$VICT, JSS8_D$KUSIV, use =
"pairwise.complete.obs")[["estimate"]][["cor"]])
cor.test(JSS8_D$OBSE, JSS8_D$KUSIV, use = "pairwise.complete.obs")
D_OBSE_cor <- c(D_OBSE_cor, cor.test(JSS8_D$OBSE, JSS8_D$KUSIV, use =
"pairwise.complete.obs")[["estimate"]][["cor"]])
cor.test(JSS8_D$BENE, JSS8_D$KUSIV, use = "pairwise.complete.obs")
D_BENE_cor <- c(D_BENE_cor, cor.test(JSS8_D$BENE, JSS8_D$KUSIV, use =
"pairwise.complete.obs")[["estimate"]][["cor"]])
cor.test(JSS8_D$OFFE, JSS8_D$KUSIV, use = "pairwise.complete.obs")
D_OFFE_cor <- c(D_OFFE_cor, cor.test(JSS8_D$OFFE, JSS8_D$KUSIV, use =
"pairwise.complete.obs")[["estimate"]][["cor"]])

#################
#Optimism (SOP2)
#################
JSS8_D$SOP <- (JSS8_D$PESS1R + JSS8_D$OPTI1)/2

describe(JSS8_D$SOP)
mvn(data = JSS8_D[, c("PESS1R", "OPTI1")], mvnTest = "hz")$multivariateNormality

SOP_MM <- 'LV =~ c(a)*PESS1R+c(a)*OPTI1

        PESS1R ~ 0*1
        LV ~ NA*1'

SOP_MM.fit_D <- sem(SOP_MM, data = JSS8_D, estimator = "mlr", missing = "fiml", std.lv =
FALSE)
semTools::reliability(SOP_MM.fit_D)

cor.test(JSS8_D$VICT, JSS8_D$SOP, use = "pairwise.complete.obs")
D_VICT_cor <- c(D_VICT_cor, cor.test(JSS8_D$VICT, JSS8_D$SOP, use =
"pairwise.complete.obs")[["estimate"]][["cor"]])
cor.test(JSS8_D$OBSE, JSS8_D$SOP, use = "pairwise.complete.obs")
D_OBSE_cor <- c(D_OBSE_cor, cor.test(JSS8_D$OBSE, JSS8_D$SOP, use =
"pairwise.complete.obs")[["estimate"]][["cor"]])
cor.test(JSS8_D$BENE, JSS8_D$SOP, use = "pairwise.complete.obs")
D_BENE_cor <- c(D_BENE_cor, cor.test(JSS8_D$BENE, JSS8_D$SOP, use =
"pairwise.complete.obs")[["estimate"]][["cor"]])
cor.test(JSS8_D$OFFE, JSS8_D$SOP, use = "pairwise.complete.obs")
D_OFFE_cor <- c(D_OFFE_cor, cor.test(JSS8_D$OFFE, JSS8_D$SOP, use =
"pairwise.complete.obs")[["estimate"]][["cor"]])

#################################
#General life satisfaction (L-1)
```

```
################################
describe(JSS8_D$LISA1)

cor.test(JSS8_D$VICT, JSS8_D$LISA1, use = "pairwise.complete.obs")
D_VICT_cor <- c(D_VICT_cor, cor.test(JSS8_D$VICT, JSS8_D$LISA1, use =
"pairwise.complete.obs")[["estimate"]][["cor"]])
cor.test(JSS8_D$OBSE, JSS8_D$LISA1, use = "pairwise.complete.obs")
D_OBSE_cor <- c(D_OBSE_cor, cor.test(JSS8_D$OBSE, JSS8_D$LISA1, use =
"pairwise.complete.obs")[["estimate"]][["cor"]])
cor.test(JSS8_D$BENE, JSS8_D$LISA1, use = "pairwise.complete.obs")
D_BENE_cor <- c(D_BENE_cor, cor.test(JSS8_D$BENE, JSS8_D$LISA1, use =
"pairwise.complete.obs")[["estimate"]][["cor"]])
cor.test(JSS8_D$OFFE, JSS8_D$LISA1, use = "pairwise.complete.obs")
D_OFFE_cor <- c(D_OFFE_cor, cor.test(JSS8_D$OFFE, JSS8_D$LISA1, use =
"pairwise.complete.obs")[["estimate"]][["cor"]])

###########################
#Left-Right self-placement
###########################
describe(JSS8_D$LRPL1)

cor.test(JSS8_D$VICT, JSS8_D$LRPL1, use = "pairwise.complete.obs")
cor.test(JSS8_D$VICT, JSS8_D$LRPL1, use = "pairwise.complete.obs")$p.value # p-value below
0.05?
D_VICT_cor <- c(D_VICT_cor, cor.test(JSS8_D$VICT, JSS8_D$LRPL1, use =
"pairwise.complete.obs")[["estimate"]][["cor"]])
cor.test(JSS8_D$OBSE, JSS8_D$LRPL1, use = "pairwise.complete.obs")
D_OBSE_cor <- c(D_OBSE_cor, cor.test(JSS8_D$OBSE, JSS8_D$LRPL1, use =
"pairwise.complete.obs")[["estimate"]][["cor"]])
cor.test(JSS8_D$BENE, JSS8_D$LRPL1, use = "pairwise.complete.obs")
D_BENE_cor <- c(D_BENE_cor, cor.test(JSS8_D$BENE, JSS8_D$LRPL1, use =
"pairwise.complete.obs")[["estimate"]][["cor"]])
cor.test(JSS8_D$OFFE, JSS8_D$LRPL1, use = "pairwise.complete.obs")
D_OFFE_cor <- c(D_OFFE_cor, cor.test(JSS8_D$OFFE, JSS8_D$LRPL1, use =
"pairwise.complete.obs")[["estimate"]][["cor"]])

##########################
#Authoritarianism (KSA-3)
##########################
JSS8_D$AAGG <- (JSS8_D$AAGG1+JSS8_D$AAGG2+JSS8_D$AAGG3+JSS8_D$ASUB1)/4
JSS8_D$ASUB <- (JSS8_D$ASUB2+JSS8_D$ASUB3)/2
JSS8_D$CONV <- (JSS8_D$CONV1+JSS8_D$CONV2+JSS8_D$CONV3)/3

describe(JSS8_D$AAGG)
mvn(data = JSS8_D[, c("AAGG1", "AAGG2", "AAGG3", "ASUB1")], mvnTest =
"hz")$multivariateNormality
describe(JSS8_D$ASUB)
mvn(data = JSS8_D[, c("ASUB2", "ASUB3")], mvnTest = "hz")$multivariateNormality
describe(JSS8_D$CONV)
mvn(data = JSS8_D[, c("CONV1", "CONV2", "CONV3")], mvnTest = "hz")$multivariateNormality

KSA_MM <- 'LV_AAGG =~ AAGG1+AAGG2+AAGG3+ASUB1
        LV_ASUB =~ c(a)*ASUB2+c(a)*ASUB3
        LV_CONV =~ CONV1+CONV2+CONV3
```

```
	AAGG1+ASUB2+CONV1 ~ 0*1
	LV_AAGG+LV_ASUB+LV_CONV ~ NA*1'

KSA_MM.fit_D <- sem(KSA_MM, data = JSS8_D, estimator = "mlr", missing = "fiml", std.lv =
FALSE)
semTools::reliability(KSA_MM.fit_D)

cor.test(JSS8_D$VICT, JSS8_D$AAGG, use = "pairwise.complete.obs")
D_VICT_cor <- c(D_VICT_cor, cor.test(JSS8_D$VICT, JSS8_D$AAGG, use =
"pairwise.complete.obs")[["estimate"]][["cor"]])
cor.test(JSS8_D$OBSE, JSS8_D$AAGG, use = "pairwise.complete.obs")
D_OBSE_cor <- c(D_OBSE_cor, cor.test(JSS8_D$OBSE, JSS8_D$AAGG, use =
"pairwise.complete.obs")[["estimate"]][["cor"]])
cor.test(JSS8_D$BENE, JSS8_D$AAGG, use = "pairwise.complete.obs")
D_BENE_cor <- c(D_BENE_cor, cor.test(JSS8_D$BENE, JSS8_D$AAGG, use =
"pairwise.complete.obs")[["estimate"]][["cor"]])
cor.test(JSS8_D$OFFE, JSS8_D$AAGG, use = "pairwise.complete.obs")
D_OFFE_cor <- c(D_OFFE_cor, cor.test(JSS8_D$OFFE, JSS8_D$AAGG, use =
"pairwise.complete.obs")[["estimate"]][["cor"]])

cor.test(JSS8_D$VICT, JSS8_D$ASUB, use = "pairwise.complete.obs")
D_VICT_cor <- c(D_VICT_cor, cor.test(JSS8_D$VICT, JSS8_D$ASUB, use =
"pairwise.complete.obs")[["estimate"]][["cor"]])
cor.test(JSS8_D$OBSE, JSS8_D$ASUB, use = "pairwise.complete.obs")
D_OBSE_cor <- c(D_OBSE_cor, cor.test(JSS8_D$OBSE, JSS8_D$ASUB, use =
"pairwise.complete.obs")[["estimate"]][["cor"]])
cor.test(JSS8_D$BENE, JSS8_D$ASUB, use = "pairwise.complete.obs")
D_BENE_cor <- c(D_BENE_cor, cor.test(JSS8_D$BENE, JSS8_D$ASUB, use =
"pairwise.complete.obs")[["estimate"]][["cor"]])
cor.test(JSS8_D$OFFE, JSS8_D$ASUB, use = "pairwise.complete.obs")
D_OFFE_cor <- c(D_OFFE_cor, cor.test(JSS8_D$OFFE, JSS8_D$ASUB, use =
"pairwise.complete.obs")[["estimate"]][["cor"]])

cor.test(JSS8_D$VICT, JSS8_D$CONV, use = "pairwise.complete.obs")
D_VICT_cor <- c(D_VICT_cor, cor.test(JSS8_D$VICT, JSS8_D$CONV, use =
"pairwise.complete.obs")[["estimate"]][["cor"]])
cor.test(JSS8_D$OBSE, JSS8_D$CONV, use = "pairwise.complete.obs")
D_OBSE_cor <- c(D_OBSE_cor, cor.test(JSS8_D$OBSE, JSS8_D$CONV, use =
"pairwise.complete.obs")[["estimate"]][["cor"]])
cor.test(JSS8_D$BENE, JSS8_D$CONV, use = "pairwise.complete.obs")
D_BENE_cor <- c(D_BENE_cor, cor.test(JSS8_D$BENE, JSS8_D$CONV, use =
"pairwise.complete.obs")[["estimate"]][["cor"]])
cor.test(JSS8_D$OFFE, JSS8_D$CONV, use = "pairwise.complete.obs")
D_OFFE_cor <- c(D_OFFE_cor, cor.test(JSS8_D$OFFE, JSS8_D$CONV, use =
"pairwise.complete.obs")[["estimate"]][["cor"]])

##############################
#Social desirability (KSE-G)
##############################
JSS8_D$SDPQ <- (JSS8_D$SDPQ1+JSS8_D$SDPQ2+JSS8_D$SDPQ3)/3
JSS8_D$SDNQ <- (JSS8_D$SDNQ1+JSS8_D$SDNQ2+JSS8_D$SDNQ3)/3

describe(JSS8_D$SDPQ)
```

```
describe(JSS8_D$SDNQ)
mvn(data = JSS8_D[, c("SDPQ1", "SDPQ2", "SDPQ3")], mvnTest = "hz")$multivariateNormality
mvn(data = JSS8_D[, c("SDNQ1", "SDNQ2", "SDNQ3")], mvnTest = "hz")$multivariateNormality

KSEG_MM <- 'LV_SDPQ =~ SDPQ1+SDPQ2+SDPQ3
	LV_SDNQ =~ SDNQ1+SDNQ2+SDNQ3

	SDPQ1+SDNQ1 ~ 0*1
	LV_SDPQ+LV_SDNQ ~ NA*1'

KSEG_MM.fit_D <- sem(KSEG_MM, data = JSS8_D, estimator = "mlr", missing = "fiml", std.lv =
FALSE)
semTools::reliability(KSEG_MM.fit_D)

cor.test(JSS8_D$VICT, JSS8_D$SDPQ, use = "pairwise.complete.obs")
D_VICT_cor <- c(D_VICT_cor, cor.test(JSS8_D$VICT, JSS8_D$SDPQ, use =
"pairwise.complete.obs")[["estimate"]][["cor"]])
cor.test(JSS8_D$OBSE, JSS8_D$SDPQ, use = "pairwise.complete.obs")
D_OBSE_cor <- c(D_OBSE_cor, cor.test(JSS8_D$OBSE, JSS8_D$SDPQ, use =
"pairwise.complete.obs")[["estimate"]][["cor"]])
cor.test(JSS8_D$BENE, JSS8_D$SDPQ, use = "pairwise.complete.obs")
D_BENE_cor <- c(D_BENE_cor, cor.test(JSS8_D$BENE, JSS8_D$SDPQ, use =
"pairwise.complete.obs")[["estimate"]][["cor"]])
cor.test(JSS8_D$OFFE, JSS8_D$SDPQ, use = "pairwise.complete.obs")
D_OFFE_cor <- c(D_OFFE_cor, cor.test(JSS8_D$OFFE, JSS8_D$SDPQ, use =
"pairwise.complete.obs")[["estimate"]][["cor"]])

cor.test(JSS8_D$VICT, JSS8_D$SDNQ, use = "pairwise.complete.obs")
D_VICT_cor <- c(D_VICT_cor, cor.test(JSS8_D$VICT, JSS8_D$SDNQ, use =
"pairwise.complete.obs")[["estimate"]][["cor"]])
cor.test(JSS8_D$OBSE, JSS8_D$SDNQ, use = "pairwise.complete.obs")
D_OBSE_cor <- c(D_OBSE_cor, cor.test(JSS8_D$OBSE, JSS8_D$SDNQ, use =
"pairwise.complete.obs")[["estimate"]][["cor"]])
cor.test(JSS8_D$BENE, JSS8_D$SDNQ, use = "pairwise.complete.obs")
D_BENE_cor <- c(D_BENE_cor, cor.test(JSS8_D$BENE, JSS8_D$SDNQ, use =
"pairwise.complete.obs")[["estimate"]][["cor"]])
cor.test(JSS8_D$OFFE, JSS8_D$SDNQ, use = "pairwise.complete.obs")
D_OFFE_cor <- c(D_OFFE_cor, cor.test(JSS8_D$OFFE, JSS8_D$SDNQ, use =
"pairwise.complete.obs")[["estimate"]][["cor"]])

########
#Health
########
describe(JSS8_D$HEAL)

cor.test(JSS8_D$VICT, JSS8_D$HEAL, use = "pairwise.complete.obs")
D_VICT_cor <- c(D_VICT_cor, cor.test(JSS8_D$VICT, JSS8_D$HEAL, use =
"pairwise.complete.obs")[["estimate"]][["cor"]])
cor.test(JSS8_D$OBSE, JSS8_D$HEAL, use = "pairwise.complete.obs")
D_OBSE_cor <- c(D_OBSE_cor, cor.test(JSS8_D$OBSE, JSS8_D$HEAL, use =
"pairwise.complete.obs")[["estimate"]][["cor"]])
cor.test(JSS8_D$BENE, JSS8_D$HEAL, use = "pairwise.complete.obs")
D_BENE_cor <- c(D_BENE_cor, cor.test(JSS8_D$BENE, JSS8_D$HEAL, use =
"pairwise.complete.obs")[["estimate"]][["cor"]])
```

```
cor.test(JSS8_D$OFFE, JSS8_D$HEAL, use = "pairwise.complete.obs")
D_OFFE_cor <- c(D_OFFE_cor, cor.test(JSS8_D$OFFE, JSS8_D$HEAL, use =
"pairwise.complete.obs")[["estimate"]][["cor"]])

###################
#Employment status
###################
#1) employed
#2) self-employed
#3) out of work and looking for work
#4) out of work but not currently looking for work
#5) doing housework
#6) pupil/student
#7) apprentice/internship
#8) retired
#[9) none of what is mentioned above]
describe(JSS8_D$EMPL)
#unemployed vs. employed
JSS8_D$EMPL.new <- recode(JSS8_D$EMPL, "1:2 = 2; 3:4 = 1; else = NA")
describe(JSS8_D$EMPL.new)

cor.test(JSS8_D$VICT, JSS8_D$EMPL.new, use = "pairwise.complete.obs")
D_VICT_cor <- c(D_VICT_cor, cor.test(JSS8_D$VICT, JSS8_D$EMPL.new, use =
"pairwise.complete.obs")[["estimate"]][["cor"]])
cor.test(JSS8_D$OBSE, JSS8_D$EMPL.new, use = "pairwise.complete.obs")
D_OBSE_cor <- c(D_OBSE_cor, cor.test(JSS8_D$OBSE, JSS8_D$EMPL.new, use =
"pairwise.complete.obs")[["estimate"]][["cor"]])
cor.test(JSS8_D$BENE, JSS8_D$EMPL.new, use = "pairwise.complete.obs")
D_BENE_cor <- c(D_BENE_cor, cor.test(JSS8_D$BENE, JSS8_D$EMPL.new, use =
"pairwise.complete.obs")[["estimate"]][["cor"]])
cor.test(JSS8_D$OFFE, JSS8_D$EMPL.new, use = "pairwise.complete.obs")
D_OFFE_cor <- c(D_OFFE_cor, cor.test(JSS8_D$OFFE, JSS8_D$EMPL.new, use =
"pairwise.complete.obs")[["estimate"]][["cor"]])

########
#Income
########
describe(JSS8_D$INCO)

cor.test(JSS8_D$VICT, JSS8_D$INCO, use = "pairwise.complete.obs")
D_VICT_cor <- c(D_VICT_cor, cor.test(JSS8_D$VICT, JSS8_D$INCO, use =
"pairwise.complete.obs")[["estimate"]][["cor"]])
cor.test(JSS8_D$OBSE, JSS8_D$INCO, use = "pairwise.complete.obs")
D_OBSE_cor <- c(D_OBSE_cor, cor.test(JSS8_D$OBSE, JSS8_D$INCO, use =
"pairwise.complete.obs")[["estimate"]][["cor"]])
cor.test(JSS8_D$BENE, JSS8_D$INCO, use = "pairwise.complete.obs")
D_BENE_cor <- c(D_BENE_cor, cor.test(JSS8_D$BENE, JSS8_D$INCO, use =
"pairwise.complete.obs")[["estimate"]][["cor"]])
cor.test(JSS8_D$OFFE, JSS8_D$INCO, use = "pairwise.complete.obs")
D_OFFE_cor <- c(D_OFFE_cor, cor.test(JSS8_D$OFFE, JSS8_D$INCO, use =
"pairwise.complete.obs")[["estimate"]][["cor"]])

#######################
#Educational attainment
```

```
########################
describe(JSS8_D$SCHO)

cor.test(JSS8_D$VICT, JSS8_D$SCHO, use = "pairwise.complete.obs")
D_VICT_cor <- c(D_VICT_cor, cor.test(JSS8_D$VICT, JSS8_D$SCHO, use =
"pairwise.complete.obs")[["estimate"]][["cor"]])
cor.test(JSS8_D$OBSE, JSS8_D$SCHO, use = "pairwise.complete.obs")
D_OBSE_cor <- c(D_OBSE_cor, cor.test(JSS8_D$OBSE, JSS8_D$SCHO, use =
"pairwise.complete.obs")[["estimate"]][["cor"]])
cor.test(JSS8_D$BENE, JSS8_D$SCHO, use = "pairwise.complete.obs")
D_BENE_cor <- c(D_BENE_cor, cor.test(JSS8_D$BENE, JSS8_D$SCHO, use =
"pairwise.complete.obs")[["estimate"]][["cor"]])
cor.test(JSS8_D$OFFE, JSS8_D$SCHO, use = "pairwise.complete.obs")
D_OFFE_cor <- c(D_OFFE_cor, cor.test(JSS8_D$OFFE, JSS8_D$SCHO, use =
"pairwise.complete.obs")[["estimate"]][["cor"]])

#####
#Age
#####
describe(JSS8_D$AGE)

cor.test(JSS8_D$VICT, JSS8_D$AGE, use = "pairwise.complete.obs")
D_VICT_cor <- c(D_VICT_cor, cor.test(JSS8_D$VICT, JSS8_D$AGE, use =
"pairwise.complete.obs")[["estimate"]][["cor"]])
cor.test(JSS8_D$OBSE, JSS8_D$AGE, use = "pairwise.complete.obs")
D_OBSE_cor <- c(D_OBSE_cor, cor.test(JSS8_D$OBSE, JSS8_D$AGE, use =
"pairwise.complete.obs")[["estimate"]][["cor"]])
cor.test(JSS8_D$BENE, JSS8_D$AGE, use = "pairwise.complete.obs")
D_BENE_cor <- c(D_BENE_cor, cor.test(JSS8_D$BENE, JSS8_D$AGE, use =
"pairwise.complete.obs")[["estimate"]][["cor"]])
cor.test(JSS8_D$OFFE, JSS8_D$AGE, use = "pairwise.complete.obs")
D_OFFE_cor <- c(D_OFFE_cor, cor.test(JSS8_D$OFFE, JSS8_D$AGE, use =
"pairwise.complete.obs")[["estimate"]][["cor"]])

########
#Gender
########
describe(JSS8_D$SEX)

cor.test(JSS8_D$VICT, JSS8_D$SEX, use = "pairwise.complete.obs")
D_VICT_cor <- c(D_VICT_cor, cor.test(JSS8_D$VICT, JSS8_D$SEX, use =
"pairwise.complete.obs")[["estimate"]][["cor"]])
cor.test(JSS8_D$OBSE, JSS8_D$SEX, use = "pairwise.complete.obs")
D_OBSE_cor <- c(D_OBSE_cor, cor.test(JSS8_D$OBSE, JSS8_D$SEX, use =
"pairwise.complete.obs")[["estimate"]][["cor"]])
cor.test(JSS8_D$BENE, JSS8_D$SEX, use = "pairwise.complete.obs")
D_BENE_cor <- c(D_BENE_cor, cor.test(JSS8_D$BENE, JSS8_D$SEX, use =
"pairwise.complete.obs")[["estimate"]][["cor"]])
cor.test(JSS8_D$OFFE, JSS8_D$SEX, use = "pairwise.complete.obs")
D_OFFE_cor <- c(D_OFFE_cor, cor.test(JSS8_D$OFFE, JSS8_D$SEX, use =
"pairwise.complete.obs")[["estimate"]][["cor"]])

##########################################################################
```

```
###Similarity of correlations across factors within countries

##UK

#Victim Sensitivity x Observer Sensitivity
VICTOBSE.cor <- data.frame(matrix(data=c(UK_VICT_cor,UK_OBSE_cor),
nrow=length(dimension.matrix[[1]]), ncol=2, dimnames = dimension.matrix))
colnames(VICTOBSE.cor) <- c("VICT", "OBSE")
cor.test(VICTOBSE.cor$VICT, VICTOBSE.cor$OBSE, use = "pairwise.complete.obs")

#Victim Sensitivity x Beneficiary Sensitivity
VICTBENE.cor <- data.frame(matrix(data=c(UK_VICT_cor,UK_BENE_cor),
nrow=length(dimension.matrix[[1]]), ncol=2, dimnames = dimension.matrix))
colnames(VICTBENE.cor) <- c("VICT", "BENE")
cor.test(VICTBENE.cor$VICT, VICTBENE.cor$BENE, use = "pairwise.complete.obs")

#Victim Sensitivity x Perpetrator Sensitivity
VICTOFFE.cor <- data.frame(matrix(data=c(UK_VICT_cor,UK_OFFE_cor),
nrow=length(dimension.matrix[[1]]), ncol=2, dimnames = dimension.matrix))
colnames(VICTOFFE.cor) <- c("VICT", "OFFE")
cor.test(VICTOFFE.cor$VICT, VICTOFFE.cor$OFFE, use = "pairwise.complete.obs")

#Observer Sensitivity x Beneficiary Sensitivity
OBSEBENE.cor <- data.frame(matrix(data=c(UK_OBSE_cor,UK_BENE_cor),
nrow=length(dimension.matrix[[1]]), ncol=2, dimnames = dimension.matrix))
colnames(OBSEBENE.cor) <- c("OBSE", "BENE")
cor.test(OBSEBENE.cor$OBSE, OBSEBENE.cor$BENE, use = "pairwise.complete.obs")

#Observer Sensitivity x Perpetrator Sensitivity
OBSEOFFE.cor <- data.frame(matrix(data=c(UK_OBSE_cor,UK_OFFE_cor),
nrow=length(dimension.matrix[[1]]), ncol=2, dimnames = dimension.matrix))
colnames(OBSEOFFE.cor) <- c("OBSE", "OFFE")
cor.test(OBSEOFFE.cor$OBSE, OBSEOFFE.cor$OFFE, use = "pairwise.complete.obs")

#Beneficiary Sensitivity x Perpetrator Sensitivity
BENEOFFE.cor <- data.frame(matrix(data=c(UK_BENE_cor,UK_OFFE_cor),
nrow=length(dimension.matrix[[1]]), ncol=2, dimnames = dimension.matrix))
colnames(BENEOFFE.cor) <- c("BENE", "OFFE")
cor.test(BENEOFFE.cor$BENE, BENEOFFE.cor$OFFE, use = "pairwise.complete.obs")

##########################################################################

##Germany

#Victim Sensitivity x Observer Sensitivity
VICTOBSE.cor <- data.frame(matrix(data=c(D_VICT_cor,D_OBSE_cor),
nrow=length(dimension.matrix[[1]]), ncol=2, dimnames = dimension.matrix))
colnames(VICTOBSE.cor) <- c("VICT", "OBSE")
cor.test(VICTOBSE.cor$VICT, VICTOBSE.cor$OBSE, use = "pairwise.complete.obs")

#Victim Sensitivity x Beneficiary Sensitivity
VICTBENE.cor <- data.frame(matrix(data=c(D_VICT_cor,D_BENE_cor),
nrow=length(dimension.matrix[[1]]), ncol=2, dimnames = dimension.matrix))
colnames(VICTBENE.cor) <- c("VICT", "BENE")
```

```
cor.test(VICTBENE.cor$VICT, VICTBENE.cor$BENE, use = "pairwise.complete.obs")

#Victim Sensitivity x Perpetrator Sensitivity
VICTOFFE.cor <- data.frame(matrix(data=c(D_VICT_cor,D_OFFE_cor),
nrow=length(dimension.matrix[[1]]), ncol=2, dimnames = dimension.matrix))
colnames(VICTOFFE.cor) <- c("VICT", "OFFE")
cor.test(VICTOFFE.cor$VICT, VICTOFFE.cor$OFFE, use = "pairwise.complete.obs")

#Observer Sensitivity x Beneficiary Sensitivity
OBSEBENE.cor <- data.frame(matrix(data=c(D_OBSE_cor,D_BENE_cor),
nrow=length(dimension.matrix[[1]]), ncol=2, dimnames = dimension.matrix))
colnames(OBSEBENE.cor) <- c("OBSE", "BENE")
cor.test(OBSEBENE.cor$OBSE, OBSEBENE.cor$BENE, use = "pairwise.complete.obs")

#Observer Sensitivity x Perpetrator Sensitivity
OBSEOFFE.cor <- data.frame(matrix(data=c(D_OBSE_cor,D_OFFE_cor),
nrow=length(dimension.matrix[[1]]), ncol=2, dimnames = dimension.matrix))
colnames(OBSEOFFE.cor) <- c("OBSE", "OFFE")
cor.test(OBSEOFFE.cor$OBSE, OBSEOFFE.cor$OFFE, use = "pairwise.complete.obs")

#Beneficiary Sensitivity x Perpetrator Sensitivity
BENEOFFE.cor <- data.frame(matrix(data=c(D_BENE_cor,D_OFFE_cor),
nrow=length(dimension.matrix[[1]]), ncol=2, dimnames = dimension.matrix))
colnames(BENEOFFE.cor) <- c("BENE", "OFFE")
cor.test(BENEOFFE.cor$BENE, BENEOFFE.cor$OFFE, use = "pairwise.complete.obs")
cor.test(BENEOFFE.cor$BENE, BENEOFFE.cor$OFFE, use = "pairwise.complete.obs")$p.value # p-
value below 0.05?

##########################################################################

###Similarity of correlations across countries

#Victim Sensitivity
VICT.cor <- data.frame(matrix(data=c(UK_VICT_cor,D_VICT_cor),
nrow=length(dimension.matrix[[1]]), ncol=2, dimnames = dimension.matrix))
cor.test(VICT.cor$UK, VICT.cor$D, use = "pairwise.complete.obs")

#Observer Sensitivity
OBSE.cor <- data.frame(matrix(data=c(UK_OBSE_cor,D_OBSE_cor),
nrow=length(dimension.matrix[[1]]), ncol=2, dimnames = dimension.matrix))
cor.test(OBSE.cor$UK, OBSE.cor$D, use = "pairwise.complete.obs")

#Beneficiary sensitivity
BENE.cor <- data.frame(matrix(data=c(UK_BENE_cor,D_BENE_cor),
nrow=length(dimension.matrix[[1]]), ncol=2, dimnames = dimension.matrix))
cor.test(BENE.cor$UK, BENE.cor$D, use = "pairwise.complete.obs")

#Perpetrator sensitivity
OFFE.cor <- data.frame(matrix(data=c(UK_OFFE_cor,D_OFFE_cor),
nrow=length(dimension.matrix[[1]]), ncol=2, dimnames = dimension.matrix))
cor.test(OFFE.cor$UK, OFFE.cor$D, use = "pairwise.complete.obs")

#################################
#Step 5: Measurement invariance
```

```
###############################

#Configural invariance
JSS8_MM_tau1 <- 'LV_VICT =~ c(a1, a1)*VICT1 + c(NA, NA)*VICT2
		LV_OBSE =~ c(a2, a2)*OBSE1 + c(NA, NA)*OBSE2
		LV_BENE =~ c(a3, a3)*BENE1 + c(NA, NA)*BENE2
		LV_OFFE =~ c(a4, a4)*OFFE1 + c(NA, NA)*OFFE2

		VICT1+OBSE1+BENE1+OFFE1 ~ c(0, 0)*1
		LV_VICT+LV_OBSE+LV_BENE+LV_OFFE ~ c(NA, NA)*1'

JSS8.fit1 <- sem(JSS8_MM_tau1, data = JSS8, group = "COUN", estimator = "mlr", missing = "fiml",
std.lv = FALSE)
summary(JSS8.fit1, standardized = T, fit.measures = T)
#--> negative residual variance // re-estimate model

#Configural invariance
JSS8_MM_tau1 <- 'LV_VICT =~ c(a1, a1)*VICT1 + c(NA, NA)*VICT2
		LV_OBSE =~ c(a2, a2)*OBSE1 + c(NA, NA)*OBSE2
		LV_BENE =~ c(a3, a3)*BENE1 + c(NA, NA)*BENE2
		LV_OFFE =~ c(a4, a4)*OFFE1 + c(NA, NA)*OFFE2

		VICT1+OBSE1+BENE1+OFFE1 ~ c(0, 0)*1
		LV_VICT+LV_OBSE+LV_BENE+LV_OFFE ~ c(NA, NA)*1

		OBSE2 ~~ c(b1, b2)*OBSE2
		b2 > 0'

JSS8.fit1 <- sem(JSS8_MM_tau1, data = JSS8, group = "COUN", estimator = "mlr", missing = "fiml",
std.lv = FALSE)
summary(JSS8.fit1, standardized = T, fit.measures = T)

#Metric invariance
JSS8_MM_tau2 <- 'LV_VICT =~ c(a1, a1)*VICT1 + c(a1, a1)*VICT2
		LV_OBSE =~ c(a2, a2)*OBSE1 + c(a2, a2)*OBSE2
		LV_BENE =~ c(a3, a3)*BENE1 + c(a3, a3)*BENE2
		LV_OFFE =~ c(a4, a4)*OFFE1 + c(a4, a4)*OFFE2

		VICT1+OBSE1+BENE1+OFFE1 ~ c(0, 0)*1
		LV_VICT+LV_OBSE+LV_BENE+LV_OFFE ~ c(NA, NA)*1'

JSS8.fit2 <- cfa(JSS8_MM_tau2, data = JSS8, group = "COUN", estimator = "mlr", missing = "fiml",
group.equal = c("loadings"), std.lv=FALSE)
summary(JSS8.fit2, standardized = T, fit.measures = T)
anova(JSS8.fit2, JSS8.fit1)

#Scalar invariance
JSS8_MM_tau3 <- 'LV_VICT =~ c(a1, a1)*VICT1 + c(a1, a1)*VICT2
		LV_OBSE =~ c(a2, a2)*OBSE1 + c(a2, a2)*OBSE2
		LV_BENE =~ c(a3, a3)*BENE1 + c(a3, a3)*BENE2
		LV_OFFE =~ c(a4, a4)*OFFE1 + c(a4, a4)*OFFE2

		VICT1+OBSE1+BENE1+OFFE1 ~ c(0, 0)*1
		LV_VICT+LV_OBSE+LV_BENE+LV_OFFE ~ c(NA, NA)*1'
```

```
JSS8.fit3 <- cfa(JSS8_MM_tau3, data = JSS8, group = "COUN", estimator = "mlr", missing = "fiml",
group.equal = c("loadings", "intercepts"), std.lv=FALSE)
summary(JSS8.fit3, standardized = T, fit.measures = T)
anova(JSS8.fit3, JSS8.fit2)

#Full uniqueness invariance
JSS8_MM_tau3 <- 'LV_VICT =~ c(a1, a1)*VICT1 + c(a1, a1)*VICT2
        LV_OBSE =~ c(a2, a2)*OBSE1 + c(a2, a2)*OBSE2
        LV_BENE =~ c(a3, a3)*BENE1 + c(a3, a3)*BENE2
        LV_OFFE =~ c(a4, a4)*OFFE1 + c(a4, a4)*OFFE2

        VICT1+OBSE1+BENE1+OFFE1 ~ c(0, 0)*1
        LV_VICT+LV_OBSE+LV_BENE+LV_OFFE ~ c(NA, NA)*1 '

JSS8.fit4 <- cfa(JSS8_MM_tau3, data = JSS8, group = "COUN", estimator = "mlr", missing = "fiml",
group.equal = c("loadings", "intercepts", "residuals"), std.lv=FALSE)
summary(JSS8.fit4, standardized = T, fit.measures = T)
anova(JSS8.fit4, JSS8.fit3)

########################################################################

#########################
#Step 6: Reference values
#########################

#Quote 1: male, lower education, 18-29
#Quote 2: male, lower education, 30-49
#Quote 3: male, lower education, 50-69
#Quote 4: male, middle education, 18-29
#Quote 5: male, middle education, 30-49
#Quote 6: male, middle education, 50-69
#Quote 7: male, upper education, 18-29
#Quote 8: male, upper education, 30-49
#Quote 9: male, upper education, 50-69
#Quote 10: female, lower education, 18-29
#Quote 11: female, lower education, 30-49
#Quote 12: female, lower education, 50-69
#Quote 13: female, middle education, 18-29
#Quote 14: female, middle education, 30-49
#Quote 15: female, middle education, 50-69
#Quote 16: female, upper education, 18-29
#Quote 17: female, upper education, 30-49
#Quote 18: female, upper education, 50-69

##UK

JSS8_UK$VICT <- (JSS8_UK$VICT1+JSS8_UK$VICT2)/2
JSS8_UK$OBSE <- (JSS8_UK$OBSE1+JSS8_UK$OBSE2)/2
JSS8_UK$BENE <- (JSS8_UK$BENE1+JSS8_UK$BENE2)/2
JSS8_UK$OFFE <- (JSS8_UK$OFFE1+JSS8_UK$OFFE2)/2

AGE1_UK <- subset(JSS8_UK, AGE == 18 | AGE == 19 | AGE == 20 | AGE == 21 | AGE == 22 |
AGE == 23 | AGE == 24 | AGE == 25 | AGE == 26
```

```
              | AGE == 27 | AGE == 28 | AGE == 29)
AGE2_UK <- subset(JSS8_UK, AGE == 30 | AGE == 31 | AGE == 32 | AGE == 33 | AGE == 34 |
AGE == 35 | AGE == 36 | AGE == 37 | AGE == 38
              | AGE == 39 | AGE == 40 | AGE == 41 | AGE == 42 | AGE == 43 | AGE == 44 | AGE ==
45 | AGE == 46 | AGE == 47
              | AGE == 48 | AGE == 49)
AGE3_UK <- subset(JSS8_UK, AGE == 50 | AGE == 51 | AGE == 52 | AGE == 53 | AGE == 54 |
AGE == 55 | AGE == 56 | AGE == 57 | AGE == 58
              | AGE == 59 | AGE == 60 | AGE == 61 | AGE == 62 | AGE == 63 | AGE == 64 | AGE ==
65 | AGE == 66 | AGE == 67
              | AGE == 68 | AGE == 69)

#Victim sensitivity
tapply(JSS8_UK$VICT, JSS8_UK$SEX, describe)

AGE1_UK$VICT <- (AGE1_UK$VICT1+AGE1_UK$VICT2)/2
describe(AGE1_UK$VICT)

AGE2_UK$VICT <- (AGE2_UK$VICT1+AGE2_UK$VICT2)/2
describe(AGE2_UK$VICT)

AGE3_UK$VICT <- (AGE3_UK$VICT1+AGE3_UK$VICT2)/2
describe(AGE3_UK$VICT)

#Observer sensitivity
tapply(JSS8_UK$OBSE, JSS8_UK$SEX, describe)

AGE1_UK$OBSE <- (AGE1_UK$OBSE1+AGE1_UK$OBSE2)/2
describe(AGE1_UK$OBSE)

AGE2_UK$OBSE <- (AGE2_UK$OBSE1+AGE2_UK$OBSE2)/2
describe(AGE2_UK$OBSE)

AGE3_UK$OBSE <- (AGE3_UK$OBSE1+AGE3_UK$OBSE2)/2
describe(AGE3_UK$OBSE)

#Beneficiary sensitivity
tapply(JSS8_UK$BENE, JSS8_UK$SEX, describe)

AGE1_UK$BENE <- (AGE1_UK$BENE1+AGE1_UK$BENE2)/2
describe(AGE1_UK$BENE)

AGE2_UK$BENE <- (AGE2_UK$BENE1+AGE2_UK$BENE2)/2
describe(AGE2_UK$BENE)

AGE3_UK$BENE <- (AGE3_UK$BENE1+AGE3_UK$BENE2)/2
describe(AGE3_UK$BENE)

#Offender sensitivity
tapply(JSS8_UK$OFFE, JSS8_UK$SEX, describe)

AGE1_UK$OFFE <- (AGE1_UK$OFFE1+AGE1_UK$OFFE2)/2
describe(AGE1_UK$OFFE)
```

```
AGE2_UK$OFFE <- (AGE2_UK$OFFE1+AGE2_UK$OFFE2)/2
describe(AGE2_UK$OFFE)

AGE3_UK$OFFE <- (AGE3_UK$OFFE1+AGE3_UK$OFFE2)/2
describe(AGE3_UK$OFFE)

##########################################################################

##Germany

JSS8_D$VICT <- (JSS8_D$VICT1+JSS8_D$VICT2)/2
JSS8_D$OBSE <- (JSS8_D$OBSE1+JSS8_D$OBSE2)/2
JSS8_D$BENE <- (JSS8_D$BENE1+JSS8_D$BENE2)/2
JSS8_D$OFFE <- (JSS8_D$OFFE1+JSS8_D$OFFE2)/2

AGE1_D <- subset(JSS8_D, AGE == 18 | AGE == 19 | AGE == 20 | AGE == 21 | AGE == 22 | AGE
== 23 | AGE == 24 | AGE == 25 | AGE == 26
                 | AGE == 27 | AGE == 28 | AGE == 29)
AGE2_D <- subset(JSS8_D, AGE == 30 | AGE == 31 | AGE == 32 | AGE == 33 | AGE == 34 | AGE
== 35 | AGE == 36 | AGE == 37 | AGE == 38
                 | AGE == 39 | AGE == 40 | AGE == 41 | AGE == 42 | AGE == 43 | AGE == 44 | AGE ==
45 | AGE == 46 | AGE == 47
                 | AGE == 48 | AGE == 49)
AGE3_D <- subset(JSS8_D, AGE == 50 | AGE == 51 | AGE == 52 | AGE == 53 | AGE == 54 | AGE
== 55 | AGE == 56 | AGE == 57 | AGE == 58
                 | AGE == 59 | AGE == 60 | AGE == 61 | AGE == 62 | AGE == 63 | AGE == 64 | AGE ==
65 | AGE == 66 | AGE == 67
                 | AGE == 68 | AGE == 69)

#Victim sensitivity
tapply(JSS8_D$VICT, JSS8_D$SEX, describe)

AGE1_D$VICT <- (AGE1_D$VICT1+AGE1_D$VICT2)/2
describe(AGE1_D$VICT)

AGE2_D$VICT <- (AGE2_D$VICT1+AGE2_D$VICT2)/2
describe(AGE2_D$VICT)

AGE3_D$VICT <- (AGE3_D$VICT1+AGE3_D$VICT2)/2
describe(AGE3_D$VICT)

#Observer sensitivity
tapply(JSS8_D$OBSE, JSS8_D$SEX, describe)

AGE1_D$OBSE <- (AGE1_D$OBSE1+AGE1_D$OBSE2)/2
describe(AGE1_D$OBSE)

AGE2_D$OBSE <- (AGE2_D$OBSE1+AGE2_D$OBSE2)/2
describe(AGE2_D$OBSE)

AGE3_D$OBSE <- (AGE3_D$OBSE1+AGE3_D$OBSE2)/2
describe(AGE3_D$OBSE)

#Beneficiary sensitivity
```

```
tapply(JSS8_D$BENE, JSS8_D$SEX, describe)

AGE1_D$BENE <- (AGE1_D$BENE1+AGE1_D$BENE2)/2
describe(AGE1_D$BENE)

AGE2_D$BENE <- (AGE2_D$BENE1+AGE2_D$BENE2)/2
describe(AGE2_D$BENE)

AGE3_D$BENE <- (AGE3_D$BENE1+AGE3_D$BENE2)/2
describe(AGE3_D$BENE)

#Offender sensitivity
tapply(JSS8_D$OFFE, JSS8_D$SEX, describe)

AGE1_D$OFFE <- (AGE1_D$OFFE1+AGE1_D$OFFE2)/2
describe(AGE1_D$OFFE)

AGE2_D$OFFE <- (AGE2_D$OFFE1+AGE2_D$OFFE2)/2
describe(AGE2_D$OFFE)

AGE3_D$OFFE <- (AGE3_D$OFFE1+AGE3_D$OFFE2)/2
describe(AGE3_D$OFFE)
```
