# Supplementary material for: An English-Language adaptation and validation of the Justice Sensitivity Short Scales–8 (JSS-8)
Source: PLoS One. 2023 Nov 6;18(11):e0293748. doi: 10.1371/journal.pone.0293748 (PMC10627457; doi:10.1371/journal.pone.0293748)
Supplement: S6 Appendix — (PDF) [file pone.0293748.s006.pdf]

S6 Appendix: Reference Ranges

Reference Ranges of the JSS-8/USS-8 Scale Scores Separately by Sex and Age Groups

	M		*SD*		Skewness		Kurtosis	
	UK	DE	UK	DE	UK	DE	UK	DE
Victim sensitivity								
Male [n_{UK} = 222; n_{DE} = 237]	3.14	3.54	1.38	1.34	0.23	−0.03	−0.78	−0.62
Female [n_{UK} = 246; n_{DE} = 237]	3.13	3.55	1.47	1.34	0.14	0.07	−0.91	−0.68
18−29 [n_{UK} = 104; n_{DE} = 105]	3.68	3.97	1.27	1.29	−0.05	−0.21	−0.56	−0.76
30−49 [n_{UK} = 180; n_{DE} = 191]	3.35	3.57	1.42	1.32	−0.05	0.01	−0.89	−0.51
50−69 [n_{UK} = 184; n_{DE} = 178]	2.62	3.26	1.36	1.33	0.68	0.18	−0.23	−0.57
Observer sensitivity								
Male [n_{UK} = 222; n_{DE} = 237]	3.83	3.80	1.25	1.15	−0.21	−0.14	−0.39	−0.17
Female [n_{UK} = 246; n_{DE} = 237]	3.96	4.22	1.22	1.12	−0.31	−0.13	−0.59	−0.59
18−29 [n_{UK} = 104; n_{DE} = 105]	4.17	4.22	1.15	1.08	−0.18	−0.21	−0.66	−0.30
30−49 [n_{UK} = 180; n_{DE} = 191]	3.89	3.87	1.22	1.06	−0.45	−0.02	−0.15	−0.26
50−69 [n_{UK} = 184; n_{DE} = 178]	3.76	4.03	1.28	1.26	−0.08	−0.23	−0.78	−0.46
Beneficiary sensitivity								
Male [n_{UK} = 222; n_{DE} = 237]	2.86	2.73	1.39	1.26	0.31	0.34	−0.84	−0.73
Female [n_{UK} = 246; n_{DE} = 237]	2.78	2.93	1.30	1.44	0.35	0.31	−0.64	−0.86
18−29 [n_{UK} = 104; n_{DE} = 105]	3.43	3.34	1.40	1.38	−0.05	0.10	−0.82	−0.90
30−49 [n_{UK} = 180; n_{DE} = 191]	2.88	2.79	1.27	1.28	0.14	0.33	−0.77	−0.73
50−69 [n_{UK} = 184; n_{DE} = 178]	2.41	2.58	1.24	1.34	0.70	0.52	−0.21	−0.61
Perpetrator sensitivity								
Male [n_{UK} = 222; n_{DE} = 237]	3.66	3.96	1.55	1.35	−0.12	−0.30	−1.00	−0.63
Female [n_{UK} = 246; n_{DE} = 237]	3.62	4.49	1.70	1.41	−0.14	−0.75	−1.20	−0.31
18−29 [n_{UK} = 104; n_{DE} = 105]	4.06	4.26	1.53	1.35	−0.43	−0.45	−0.77	−0.68
30−49 [n_{UK} = 180; n_{DE} = 191]	3.67	4.14	1.58	1.41	−0.19	−0.36	−1.04	−0.76
50−69 [n_{UK} = 184; n_{DE} = 178]	3.38	4.29	1.68	1.43	0.11	−0.63	−1.18	−0.42

Note. UK = United Kingdom (N = 468); DE = Germany (N = 474).
